# Autophagy gene expression in skeletal muscle of older individuals is associated with physical performance, muscle volume and mitochondrial function in the study of muscle, mobility and aging (SOMMA)

**DOI:** 10.1111/acel.14118

**Published:** 2024-04-16

**Authors:** Paul M. Coen, Zhiguang Huo, Gregory J. Tranah, Haley N. Barnes, Xiping Zhang, Christopher A. Wolff, Kevin Wu, Peggy M. Cawthon, Russell T. Hepple, Frederico G. S. Toledo, Daniel S. Evans, Olaya Santiago‐Fernández, Ana Maria Cuervo, Stephen B. Kritchevsky, Anne B. Newman, Steven R. Cummings, Karyn A. Esser

**Affiliations:** ^1^ Translational Research Institute, AdventHealth Orlando Florida USA; ^2^ Department of Biostatistics, College of Public Health & Health Professions College of Medicine University of Florida Gainesville Florida USA; ^3^ California Pacific Medical Center Research Institute San Francisco California USA; ^4^ Department of Physiology and Aging, College of Medicine University of Florida Gainesville Florida USA; ^5^ Department of Epidemiology and Biostatistics University of California San Francisco San Francisco California USA; ^6^ Department of Physical Therapy University of Florida Gainesville Florida USA; ^7^ Department of Medicine, Division of Endocrinology and Metabolism University of Pittsburgh School of Medicine Pittsburgh Pennsylvania USA; ^8^ Department of Developmental & Molecular Biology Albert Einstein College of Medicine New York New York USA; ^9^ Department of Internal Medicine Wake Forest University School of Medicine Winston‐Salem North Carolina USA; ^10^ Department of Epidemiology, School of Public Health University of Pittsburgh Pittsburgh Pennsylvania USA

**Keywords:** aging, autophagy, gene expression, mitochondria, mobility, mTor, oxidative metabolism

## Abstract

Autophagy is essential for proteostasis, energetic balance, and cell defense and is a key pathway in aging. Identifying associations between autophagy gene expression patterns in skeletal muscle and physical performance outcomes would further our knowledge of mechanisms related with proteostasis and healthy aging. Muscle biopsies were obtained from participants in the Study of Muscle, Mobility, and Aging (SOMMA). For 575 participants, RNA was sequenced and expression of 281 genes related to autophagy regulation, mitophagy, and mTOR/upstream pathways was determined. Associations between gene expression and outcomes including mitochondrial respiration in muscle fiber bundles (MAX OXPHOS), physical performance (VO_2_ peak, 400 m walking speed, and leg power), and thigh muscle volume, were determined using negative binomial regression models. For autophagy, key transcriptional regulators including TFE3 and NFKB‐related genes (RELA, RELB, and NFKB1) were negatively associated with outcomes. On the contrary, regulators of oxidative metabolism that also promote overall autophagy, mitophagy, and pexophagy (PPARGC1A, PPARA, and EPAS1) were positively associated with multiple outcomes. In line with this, several mitophagy, fusion, and fission‐related genes (NIPSNAP2, DNM1L, and OPA1) were also positively associated with outcomes. For mTOR pathway and related genes, expression of WDR59 and WDR24, both subunits of GATOR2 complex (an indirect inhibitor of mTORC1), and PRKAG3, which is a regulatory subunit of AMPK, were negatively correlated with multiple outcomes. Our study identifies autophagy and selective autophagy such as mitophagy gene expression patterns in human skeletal muscle related to physical performance, muscle volume, and mitochondrial function in older persons which may lead to target identification to preserve mobility and independence.

## Graphical abstract

Our study identifies autophagy and selective autophagy (mitophagy) gene expression patterns in human skeletal muscle related to physical performance, muscle volume, and mitochondrial function in older persons.

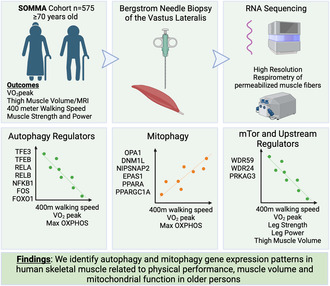

**Abbreviations**
ADP: adenosine diphosphate
AMPK: AMP‐activated protein kinase
ANOVA: analysis of variance
ATF: activating transcription factor
ATG: autophagy‐related
ATPV1B1: V‐type proton ATPase subunit B1
BECN: Beclin‐1
BMI: body mass Index
CHAMPS: community healthy activities model program for seniors
CLEAR: coordinated lysosomal expression and regulation
CPET: cardiopulmonary exercise testing
D3CR: D3‐creatine
DNM1L: Dynamin‐1‐like
ECG: electrocardiogram
EEF2K: eukaryotic elongation factor 2 kinase
EGTA: ethylene glycol tetraacetic acid
ENSG: Ensembl gene ID
EPAS: endothelial PAS domain‐containing protein 1
FOS: Fos proto‐oncogene
FOXO: forkhead box protein O
GATOR: GTPase‐activating protein toward Rags
GLM: generalized linear model
IL: interleukin
MAPKAP: MAP kinase‐activated protein
MAX OXPHOS: maximal oxidative phosphorylation
MFN: mitofusin
mTORC: mechanistic target of rapamycin complex
MUL: mitochondrial E3 ubiquitin protein ligase
MuRF: muscle RING‐finger protein
MYC: cellular myelocytomatosis
NFKB: nuclear factor kappa‐light‐chain‐enhancer of activated B cells
NIPSNAP: 4‐nitrophenylphosphatase domain and non‐neuronal SNAP25‐like 1
OPA1: optic atrophy 1 mitochondrial dynamin like GTPase
PPARA: peroxisome proliferator activated receptor alpha
PPARGC1A: peroxisome proliferator‐activated receptor gamma coactivator 1‐alpha
PRKAG: protein kinase AMP‐activated non‐catalytic subunit gamma
RCR: respiratory control ratio
REL: reticuloendotheliosis viral oncogene homolog
ROS: reactive oxygen species
SIRT: silent mating type information regulation 2 homolog
SOMMA: study of muscle, mobility and aging
TFE: transcription factor E
TNF: tumor necrosis factor
TSC: tuberous sclerosis 1
VCO_2_
: volume of carbon dioxide produced
VO_2_ peak: maximal oxygen consumption
VO_2_
: volume of oxygen consumed
WDR: WD repeat domain

## 1 INTRODUCTION

Aging is defined by a gradual loss of physiological integrity, with accrual of cellular damage widely considered the general cause. Several molecular hallmarks of aging have been proposed that contribute to accumulation of cellular damage, including genomic instability, loss of proteostasis, macroautophagy dysregulation, DNA damage, and mitochondrial dysfunction. Aging, particularly sedentary aging, results in a loss of muscle mass, strength, and oxidative capacity that contributes to lower cardiorespiratory fitness, slower walking speed, and ultimately mobility limitations. Indeed, physical activity level plays an important role in modulating these age‐associated changes (St‐Jean‐Pelletier et al., 2017). These changes in muscle are linked to impaired protein homeostasis, or proteostasis including the loss of skeletal muscle proteins and alterations in the balance between protein synthesis and degradation (Rasmussen et al., 2006). The molecular basis for these changes, including changes in the expression of several key genes and proteins implicated in growth, atrophy, proteosome degradation, and mitochondrial metabolism, is also documented (Masiero et al., 2009; Sandri, 2010).

Autophagy is the cellular process of lysosomal degradation and recycling of cytoplasmic components to maintain cellular homeostasis. Autophagy clears and recycles damaged organelles and macromolecules and preserves DNA stability (Sandri, 2010). Autophagy decreases with aging in several tissues; however, its role in regulating muscle mass and function remains poorly understood, particularly in humans. On one hand, excessive activation of autophagy aggravates muscle wasting by removing portions of cytoplasm, proteins, and organelles (Mammucari et al., 2007). Conversely, it was demonstrated that inhibition of autophagy can result in muscle degeneration and weakness (Masiero et al., 2009). Blocking autophagy in muscle can also lead to impacts on innervation, mitochondrial function, and oxidative damage (Carnio et al., 2014).

Autophagy is highly coordinated and is positively regulated by the energy sensor, AMP‐activated protein kinase (AMPK) pathway, and negatively regulated by the nutrient sensor, the mammalian target of rapamycin (mTOR) pathway (Jung et al., 2010). The transcription factors, TFEB and TFE3, are recognized for their role regulating a network of genes (the CLEAR network) involved in lysosomal biogenesis, autophagy, and lysosomal exocytosis (Sardiello et al., 2009). The nuclear localization of these transcription factors is modulated by phosphorylation via both AMPK and mTORC pathways. The study of autophagy has led to the identification of multiple autophagy sub‐types that selectively degrade organelles including mitophagy, the process of mitochondrial degradation, and pexophagy, which is the targeted degradation of peroxisomes. PGC‐1α is a regulator of mitochondrial biogenesis and has been shown to also activate mitochondria fusion‐fission events, autophagy and in particular mitophagy (Vainshtein et al., 2015). While the extensive study of the regulation of autophagic flux (the combined process of autophagosome formation and clearance) in model systems has revealed this regulation to be complex and multifactorial, the relevant pathways that regulate autophagy and mitophagy in human muscle aging have yet to be fully elucidated.

To further understand the gene expression profiles related to autophagy in human muscle and how they associate with muscle and physical function phenotypes, we performed sequencing of RNA obtained from muscle biopsies collected from participants in the Study of Muscle, Mobility, and Aging (SOMMA). SOMMA is a prospective, longitudinal study of older people at risk of major mobility disability designed to understand the contributions of skeletal muscle mass and key properties of muscle tissue from biopsies to mobility. Decreases in autophagy occur with aging and a few small studies have correlated protein markers of autophagy with compromised muscle function (Aas et al., 2019; Zeng et al., 2018). In this investigation, we examined associations between expression levels of 260 genes involved in autophagy with muscle mitochondrial function, 400‐m walking speed, VO_2_ peak, leg strength, and thigh muscle volume. Here, we took a targeted candidate gene approach and hypothesized that expression of autophagy genes in the muscle of older persons is generally associated with muscle mitochondrial function and tissues beyond muscle, thereby substantially contributing to overall fitness, walking speed, strength, and muscle mass.

## 2 RESULTS

### 2.1 Participant characteristics

**FIGURE 1:**
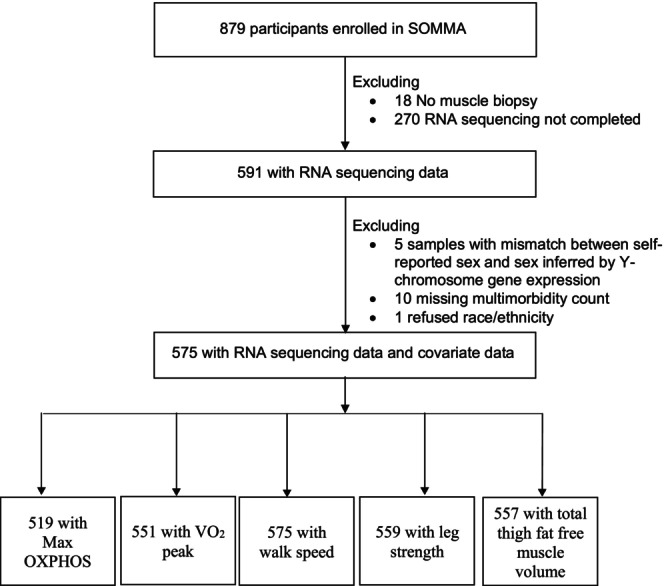
Participant inclusions. SOMMA participants selected for cross‐sectional analyses.

A total of 879 participants provided consent and completed baseline measurements across both clinical sites (Figure 1). Of the 879 participants with complete baseline measures, 591 participants had RNA sequencing completed, and 575 of these had high‐quality sequencing and complete covariate data. The characteristics of the study population with complete RNA sequencing and complete covariate data are presented in Table 1.

**TABLE 1:** Baseline characteristics of included SOMMA participants also stratified by tertiles of 400 m Walking Speed.

Variable	Total *N*	Total summary (*N* = 575)	Tertile 1, 0.458 <= T1 < 1.012 (*N* = 191)	Tertile 2, 1.012 <= T2 < 1.154 (*N* = 192)	Tertile 3, 1.154 <= T3 < 1.591 (*N* = 192)	*p*‐Value	*p*‐Trend
Clinic Site: Pittsburgh	575	263 (45.7)	93 (48.7)	76 (39.6)	94 (49.0)	0.111	0.954
Age, years	575	75.9 ± 4.5	77.5 ± 5.0	75.6 ± 4.2	74.73 ± 3.78	<0.001	<0.001
Sex: Female	575	319 (55.5)	121 (63.4)	109 (56.8)	89 (46.4)	0.003	<0.001
Race: Non‐Hispanic White	575	503 (87.5)	158 (82.7)	171 (89.1)	174 (90.6)	0.047	0.020
Height, m	575	1.7 ± 0.1	1.6 ± 0.1	1.7 ± 0.1	1.7 ± 0.1	<0.001	<0.001
Weight, kg	575	76.2 ± 15.5	79.2 ± 15.7	75.9 ± 15.3	73.4 ± 14.9	0.001	<0.001
CHAMPS: Hours/week in all exercise‐related activities	575	15.7 ± 11.4	11.4 ± 8.6	16.3 ± 12.1	19.3 ± 11.9	<0.001	<0.001
Multimorbidity Count	575					0.049	0.003
0 Chronic conditions		254 (44.2)	74 (38.7)	80 (41.7)	100 (52.1)		
1 Chronic condition		220 (38.3)	75 (39.3)	78 (40.6)	67 (34.9)		
2+ Chronic conditions		101 (17.6)	42 (22.0)	34 (17.7)	25 (13.0)		
VO_2_ peak (mL/min)	551	1564.0 ± 425.6	1392.1 ± 369.5	1574.6 ± 408.1	1710.3 ± 434.9	<0.001	<0.001
Leg Strength: 1 repetition maximum	559	177.9 ± 62.1	155.2 ± 52.6	176.5 ± 58.0	200.6 ± 66.2	<0.001	<0.001
P1‐Max OXPHOS (pmol/(s*mg))	519	61.5 ± 18.1	55.2 ± 15.1	61.4 ± 17.2	67.8 ± 19.6	<0.001	<0.001
Total Thigh Fat Free Muscle vol, L	557	9.1 ± 2.3	8.7 ± 2.2	9.1 ± 2.3	9.6 ± 2.4	<0.001	<0.001
D_3_CR muscle mass (kg)	551	22.6 ± 6.6	21.6 ± 6.1	22.8 ± 6.7	23.4 ± 6.9	0.027	0.008

*Note*: Data shown as *n* (%), mean ± Standard Deviation. *p*‐Values are presented for variables across tertiles of 400 m gait speed. *p*‐Values for continuous variables from ANOVA for normally distributed data, a Kruskal–Wallis test for skewed data. *p* for linear trend across categories was calculated with linear regression models for those normally distributed variables, a Jonckheere–Terpstra test for skewed data. *p*‐Values for categorical data from a chi‐square test for homogeneity. *p* for trend was calculated with the Jonckheere–Terpstra test.

### 2.2 RNA (human Ensembl genes [ENSG]) detection

The mean, median, and SD of the PCR duplicate percent per sample were 59%, 56%, and 9%, respectively (Table S1). After PCR duplicates were removed, the number of aligned reads per sample was high (mean = 69,117,209, median = 71,313,059, SD = 14,444,848, range = 12,853,785–102,724,183).

### 2.3 Association of autophagy gene expression with multiple outcomes

**FIGURE 2:**
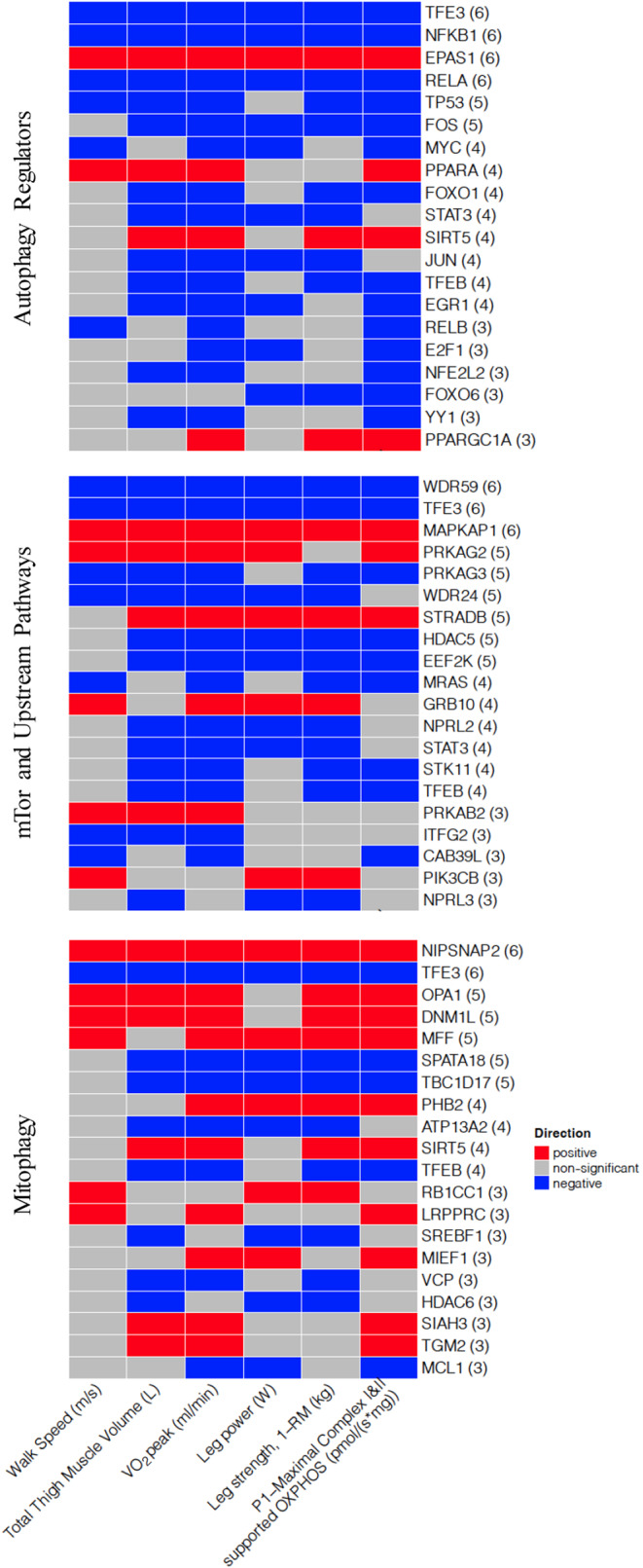
Significant associations of autophagy genes with mitochondrial function, physical performance, and muscle mass measures. Heat map capturing top 20 statistically significant (*p* < 0.05 FDR adjusted) genes identified by our models: each color represents positive (red) or negative (blue) associations.

We utilized a published, curated list of autophagy genes (Bordi et al., 2021) to conduct a targeted analysis of the RNASeq dataset. A total of 260 genes uniquely categorized to autophagy regulation (56 genes), mitophagy (64 genes), mTOR and upstream pathways (121 genes) or, a combination of the three gene sets (19 genes) were analyzed (Table S2). For each gene set, we examined the genes that were most significantly associated across multiple outcomes (Figure 2). All results report log‐base 2‐fold changes reflecting the change in gene expression per one SD unit increase in each trait.

Analysis of the curated list of autophagy genes did not reveal any significant correlations between key core autophagy machinery (BECN1, ATG7, and ATG5) and outcomes (Max OXPHOS, VO_2_ peak). For autophagy regulators, key transcriptional regulators including TFE3, TFEB, NFKB‐related genes (RELA, RELB, NFKB1), FOS, and FOXO1 were all significantly negatively associated with 400 m walk speed, VO_2_ peak, and Max OXPHOS. Alternatively, regulators of oxidative metabolism that also promote autophagy and mitophagy including PPARGC1A, PPARA, and EPAS1, a driver of pexophagy, were all positively associated with VO_2_ peak and Max OXPHOS. We also found that several mitochondria, fusion, and fission‐related genes (NIPSNAP2, DNM1L, and OPA1) were also positively associated with multiple outcomes including VO_2_ peak, Max OXPHOS and 400 m walking speed. For mTOR pathway and related genes, expression of WDR59 and WDR24, both subunits of GATOR2 complex (an inhibitor of mTORC1), and PRKAG3, which is a regulatory subunit of AMPK, were negatively associated with multiple outcomes including 400 m walk speed, VO_2_ peak, leg strength, leg power, and thigh muscle volume. In contrast, MAPKAP1, which is a subunit of mTORC2, was positively associated with outcomes. A summary of all statistically significant associations for each gene set: autophagy regulation, mitophagy, and mTOR and upstream pathways with each trait is presented in Tables S3–S8.

### 2.4 Association of mitochondrial respiration with gene expression

**FIGURE 3:**
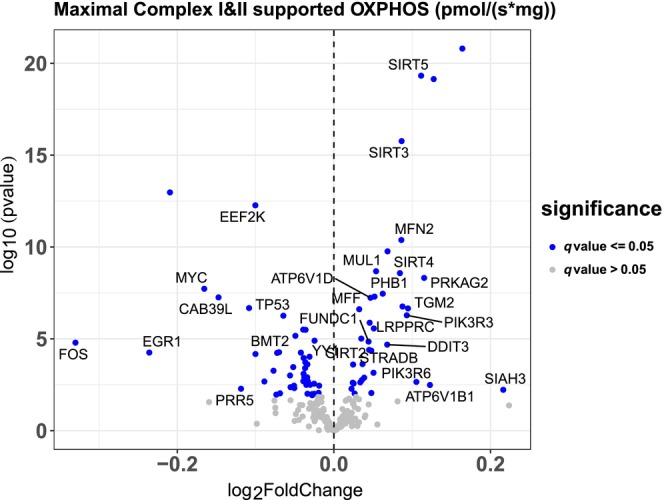
Associations with Max OXPHOS. Volcano plot capturing all statistically significant (*p* < 0.05 FDR adjusted) genes identified by our models: Each dot represents a gene; the dot color indicates significance level. Base model: gene expression~ Max OXPHOS + age + gender + clinic site + race/ethnicity + height + weight + physical activities + multimorbidity count + sequencing batch.

We next examined associations between gene expression for all three gene sets and specific outcomes. Maximal Complex I&II supported OXPHOS was positively associated with the expression of the deacetylases SIRT5 and SIRT3, the regulators of mitochondrial dynamics MFN2, MUL1, and the component of the vacuolar proton pump ATPV1B1 (Figure 3). Conversely, OXPHOS was negatively associated with the transcription factors FOS, MYC, and the regulator of protein translation EEF2K, among others. The full list of associations between autophagy genes and Max OXPHOS is presented in Table S3.

### 2.5 Association of VO_2_ peak with gene expression

**FIGURE 4:**
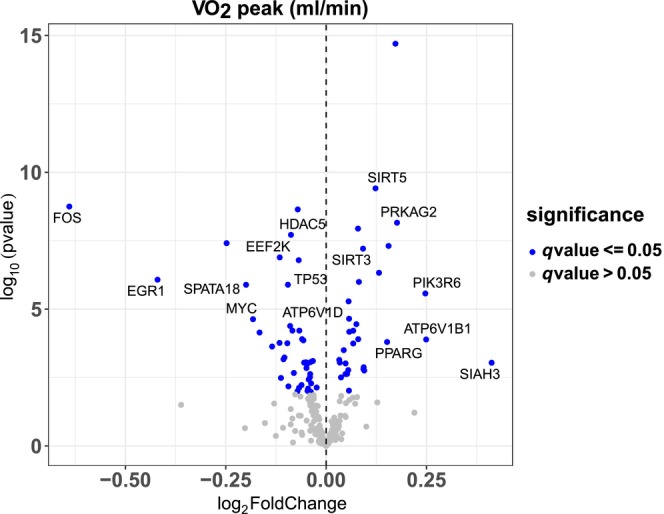
Associations with VO_2_ peak. Volcano plot capturing all statistically significant (*p* < 0.05 FDR adjusted) genes identified by our models: Each dot represents a gene; the dot color indicates significance level. Base model: gene expression~ VO_2_ Peak + age + gender + clinic site + race/ethnicity + height + weight + physical activities + multimorbidity count + sequencing batch.

Autophagy genes that were positively associated with higher VO_2_ peak, included the autophagy regulators PPARGC1A and PPARA, the mitochondria dynamic regulators related to mitophagy DNM1L and OPA1, the mitophagy core gene NIPSNAP2, and the pexophagy‐related hypoxia‐induced gene EPAS1 (Figure 4). Genes that were negatively associated with VO_2_ peak, included the autophagy/exocytosis regulators RELA, RELB, and the regulators of AMPK and mTOR, PRKAG3, and WDR24, respectively. The full list of associations between autophagy genes and Max OXPHOS is presented in Table S4.

### 2.6 Association of 400 m walk speed with gene expression

**FIGURE 5:**
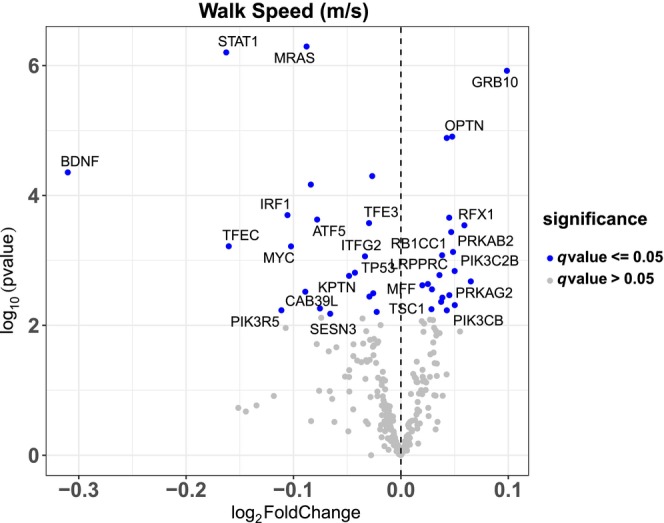
Associations with 400 m Walk Speed. Volcano plot capturing all statistically significant (*p* < 0.05 FDR adjusted) genes identified by our models: Each dot represents a gene; the dot color indicates significance level. Base model: gene expression~ walk speed + age + gender + clinic site + race/ethnicity + height + weight + physical activities + multimorbidity count + sequencing batch.

Finally, we examined associations between autophagy‐related gene expression and 400 m walk speed. Genes that were positively associated with higher 400 m walk speed included mTOR and upstream pathway genes PRKAG2, PRKAB2, and TSC1 (Figure 5). Genes that were negatively associated with 400 m walk speed included positive (TFEC, TFE3) and negative (ATF5) autophagy regulators and the mTOR regulator SESN3. The full list of associations between autophagy genes and 400 m walk speed is presented in Table S5. The list of associations between autophagy genes and leg strength, leg power, and thigh muscle volume is presented in Tables S6–S8.

## 3 DISCUSSION

Autophagy is a critical mechanism for maintaining cellular homeostasis, the dysregulation of which has been implicated as a key contributor to the biology of aging. Despite this, the relevance of skeletal muscle autophagy to measures of physical function, fitness, muscle mass, and mitochondrial function in older adults has received relatively little attention. Here, we leveraged transcriptomic data generated from muscle biopsy samples obtained from adults participating in the Study of Muscle, Mobility, and Aging (SOMMA) to investigate the relationships between autophagy gene expression and multiple aging muscle and whole‐body phenotypes (Cummings et al., 2023). We identified distinct patterns of association between expression of genes known to be key transcriptional regulators and signaling molecules of the autophagy program with several functional and physical domains. These data provide unique insight into the transcriptional regulation of autophagy in clinically relevant human aging phenotypes.

The principal finding was that several transcriptional regulators of the autophagy/lysosome pathway including TFE3, TFEB, FOS, and FOXO1 were all negatively associated with VO_2_ peak, Max OXPHOS, leg strength, and thigh muscle volume. These data were unexpected based on literature showing that autophagy decreases with aging in several tissues, but they could be reconciled when considering the contribution of posttranslational mechanisms to autophagy regulation and the fact that this is a human cross‐sectional study. Our data are congruent with reports of a dysregulated autophagy response in aging muscle, including higher expression of mediators of autophagy (O'Leary et al., 2013). In physically inactive older adults, positive correlations between autophagy mediators and longer time to complete the sit‐to‐stand test (poor function), and poor lower extremity tissue composition have been reported (Picca et al., 2023). We suggest two plausible explanations for the negative association between functional outcomes and transcriptional regulators of the autophagy/lysosome pathway. Transcriptional changes in autophagy may reflect a compensatory mechanism in response to impairment of the last steps on the autophagy process, or alternatively, the reduced transcriptional activation of autophagy in individuals with better functional outcomes could be a consequence of less need for quality control autophagy in those individuals.

In the first scenario, the negative association between functional outcomes and expression of transcriptional regulators of autophagy at the level of induction could be interpreted as a response to a failure in the autophagic process. In fact, poor clearance of already formed autophagosomes with age was one of the first defects identified in autophagy in model systems (Terman, 1995). Under these conditions, lower induction of autophagosome formation could be a protective mechanism against autophagosome accumulation in the old muscle. In support of these efforts to adjust autophagosome formation to their clearance, we found positive correlation with genes involved in the later autophagy steps such as ATPV1B1, which could contribute to increase autophagosome clearance efficiency by preserving lysosomal acidification.

Our finding that autophagy regulators RELA, RELB, and NFKB1 are negatively associated with outcomes is in line with previous data showing that NF‐kB signaling is upregulated within aged skeletal muscle (Bar‐Shai et al., 2005; Phillips & Leeuwenburgh, 2005), including a report by Buford et al. (2010), who observed higher activity of NF‐kB in older sedentary adults with low fitness, muscle mass, and strength compared to a young control group. Furthermore, RELA and RELB, as components of the exocyst complex contribute to regulate protein exocytosis, recently shown to become enhanced in response to reduced intracellular degradation with age (Krause et al., 2022). The observed negative association of RELA and RELB expression with outcomes could be an indication of the proposed bias toward extracellular release of cargo in response to reduced autophagy.

Others have shown that TFEB protein expression is not decreased in muscle with aging (Picca et al., 2023), and that exercise induces expression of TFEB (Mansueto et al., 2017). Paradoxically, TFEB appears to also be upregulated in the context of inactivity. Others have reported that chronic activation of the TFEB/MuRF1 pathway leads to skeletal muscle wasting (Du Bois et al., 2015). There are several reports indicating that a decline in mitochondrial oxidative capacity in muscle can trigger TFEB expression (Triolo, Oliveira, et al., 2022; Triolo, Slavin, et al., 2022). It may be that in unhealthy muscle aging (poor outcomes) there is a compensatory increase in gene expression of TFEB and TFE3 expression (and other autophagy regulators) in response to increased oxidative damage. Thus, the observation from SOMMA data that higher levels of TFEB and TFE3 mRNAs are negatively associated with outcomes may reflect insufficient autophagy leading to a feedback transcriptional mechanism. In line with this, we found modest or no correlation with expression of many of the core autophagy machinery. In this case, and considering the positive relationships identified for outcomes and genes that participate in mitophagy, it is possible that in a context with reduced clearance, mitochondrial turnover by autophagy is prioritized and the autophagy core machinery is preferentially allocated for execution of mitophagy. However, this is speculative, and we recognize the need for future studies gain more direct evidence for these associations.

Our results imply that older adults with higher fitness/respiration and walking speed may have lower levels of oxidative stress and inflammation and thus less need for quality control autophagy, which could explain lower expression of drivers of the autophagy program. This finding is congruent with reports that higher levels of circulating inflammatory mediators are associated with poor mobility in older adults (Penninx et al., 2004). While we have not probed markers of inflammation in this SOMMA dataset, we note that a separate analysis supports the concept that older adults with higher fitness/respiration/walking speed also have lower oxidative stress (Day et al., 2024; Tranah et al., 2023).

We found that expression of PPARA and PPARGC1A was positively associated with multiple outcomes including VO_2_ peak and Max OXPHOS. PPARGC1A, the gene that encodes PGC‐1α, is most well‐known for its role in mitochondrial biogenesis but has been reported to protect muscle mass in the context of muscle atrophy triggered by aging (Yang et al., 2020), chronic heart failure, and various other muscle wasting conditions (Sandri et al., 2006). More recently, it has been shown that PPARGC1A also plays a role in activating the autophagy‐lysosome pathway (Takikita et al., 2010) and specifically mitophagy in muscle (Vainshtein et al., 2015). In addition, PPARGC1A inhibits the expression of multiple pro‐inflammatory mediators, including TNF‐α and IL‐6, and regulates antioxidant defense mechanisms (Kang & Li Ji, 2012), both of which in turn can activate autophagy. In line with the role of PPARGC1A in mitophagy, we found that several mitochondria, fusion, and fission‐related genes (DNM1L, OPA1) and the mitophagy “eat me” signal NIPSNAP2 were also positively associated with multiple outcomes, including VO_2_ peak, Max OXPHOS, and 400 m walking speed. In rodent genetic models, these findings indicate that mitophagy, mitochondrial fusion, and fission are essential for skeletal muscle health (Dulac et al., 2020; Favaro et al., 2019; Tezze et al., 2017). However, studies in humans are equivocal on the impact of aging *per se* on Opa1 protein content in the skeletal muscle, with sedentary young and old humans having similar levels (Distefano et al., 2017), while sedentary older adults had lower expression of Opa1 protein compared to younger adults (Tezze et al., 2017). With respect to Drp1 expression, some studies report a decreased (Distefano et al., 2017) or an unchanged (Joseph et al., 2012) Drp1 content in skeletal muscle of older individuals. However, unlike the previous study designs, our analysis focuses on associations between mitophagy gene expression and age‐related phenotypes rather than aging *per se*. A majority of evidence shows that better muscle and mitochondrial health occur concomitantly with elevated markers of mitochondrial fission, mitophagy receptors, and oxidative phosphorylation in human skeletal muscle of older adults who are active (Balan et al., 2019; Drummond et al., 2014; Tezze et al., 2017) or who completed an exercise training program (Konopka et al., 2014). However, others have reported an impairment in mitophagy even in muscle from physically active older adults (Gouspillou et al., 2014). Physiological levels of mild ROS production in mitochondria play essential roles in redox signaling, including induction of mitophagy (Frank et al., 2012; Sakellariou et al., 2016), and may be a link between mitophagy gene expression and better mitochondrial and functional outcomes.

Mammalian target of rapamycin C1 (mTORC) is a master regulator of cell growth and metabolism through sensing and integrating different nutritional and environmental cues. Dysregulation and hyperactivation of mTOR contribute to several age‐related diseases, such as cancer, neurodegenerative diseases, and type 2 diabetes mellitus. In skeletal muscle, mTORC1 signaling plays a critical role in protein synthesis and degradation, including regulating many steps of autophagy. The decline in skeletal muscle mass with aging is complex, but anabolic resistance, or the limited ability to increase protein synthesis and inhibit autophagy in response to stimuli such as amino acids, is implicated as a contributing factor. In this study, we found that expression of mTOR pathway‐related genes WDR59 and WDR24 was both negatively correlated with 400 m walk speed, VO_2_ peak, leg strength, leg power, and thigh muscle volume. WDR59 and WDR24 are both subunits of GATOR2 complex, which functions as a positive regulator of amino‐acid‐mediated mTORC1 activation (Bar‐Peled et al., 2013), and WDR24 modulates mTOR through the previously described changes in lysosome cellular positioning and dynamics (Korolchuk et al., 2011) and thus indirectly modulates autophagic flux (Cai et al., 2016). This is in line with the observation that TFEB expression is also negatively associated with outcomes as mTORC1 regulates TFEB localization to the nucleus and activation by phosphorylating the transcription factor on several serine and threonine residues (Rabanal‐Ruiz et al., 2017). Taken together, the associations of WDR59, WDR24, and TFEB with outcomes suggest that greater performance/fitness in older adults could be related to a blunted capacity of muscle mTORC1 to inhibit autophagy.

Our study has limitations. The analysis was performed on whole muscle tissue, and the impact of mitochondrial content and muscle fiber type proportions on gene expression is not accounted for. For example, we observed that several genes whose expression is correlated with Max OXPHOS are expressed exclusively within (or on) the mitochondria (SIRT5, SIRT3, MFN2, MUL1). We also acknowledge that having more data including protein expression and autophagy flux measurements would provide a more complete picture of the complex regulation of autophagy within muscle of aged individuals. That said, examining relationships between gene expression and relevant aging phenotypes in whole tissue initially is important to establish proof of concept/mechanism and will inform necessary future studies. A second limitation is that the study participants are of mostly White ancestry, thus potentially limiting generalizability of findings. Our data are cross‐sectional and observational, which limits our ability to prove causality. A strength of our study is that we paired muscle gene expression and mitochondrial function with several measures of fitness, strength, and muscle mass and accounted for potential confounding factors. Previous studies did not include older adults at risk of mobility disability and only analyzed cross‐sectional associations between one or two properties and physical performance. Moreover, by focusing on a curated set of genes rather than the broader gene ontology families, we were able to test specific hypotheses regarding the role of autophagy across mitochondrial function, fitness, mobility, strength, and muscle volume.

## 4 CONCLUSION

This study of autophagy genes in 575 SOMMA participants has revealed significant associations between autophagy regulation, mitophagy, and mTOR pathways genes and a diverse range of clinically relevant phenotypes that include walking speed, VO_2_ peak, maximal mitochondrial respiration, total thigh muscle volume, leg power, and leg strength. Our results support and add to the evidence, which suggests that changes in transcriptional regulation of autophagy may contribute to changes in various indices of muscle function that are key to mobility with aging. Additional studies are needed to further decipher the role of autophagy, including the contribution of its post‐transcriptional regulation, in skeletal muscle fitness during aging.

## 5 EXPERIMENTAL PROCEDURES

### 5.1 Study population

The Study of Muscle, Mobility, and Aging (SOMMA) is a prospective cohort study of mobility in community‐dwelling older adults. Participants for the current study were from the baseline cohort, enrolled between April 2019 and December 2021 (Cummings et al., 2023). SOMMA was conducted at 2 clinical sites: University of Pittsburgh (Pittsburgh, PA) and Wake Forest University School of Medicine (Winston‐Salem, NC). Eligible participants were ≥ 70 years old at enrollment, had a body mass index (BMI) of 18–40 kg/m^2^, and were eligible for magnetic resonance (MR) imaging and a muscle tissue biopsy (Cummings et al., 2023). Individuals were further excluded if they had active cancer or were in the advanced stages of heart failure, renal failure on dialysis, dementia, or Parkinson's disease. Participants must have been able to complete the 400‐meter walk; those who appeared as they might not be able to complete the 400 m walk at the in‐person screening visit completed a short‐distance walk (4 meters) to ensure their walking speed as > = 0.6 m/s. The study protocol was approved by the Western Institutional Review Board Copernicus Group (WCG IRB; study number 20180764), and all participants provided written informed consent. In brief, baseline testing occurred across 3 separate days of clinic visits that were generally within 6–8 weeks of each other. The mean time between Day 1 and 3 was 42 days or ~ 6 weeks. Day 1 included general clinic assessments (e.g., physical and cognitive tests; 5 h), Day 2 included magnetic resonance imaging and Cardiopulmonary Exercise Testing (MR and CPET, 2–3 h), and Day 3 included fasting specimen and tissue collection (2 h). There were 879 participants who completed Day 1 of baseline testing and had at least one primary SOMMA measure: CPET, MR imaging, or muscle tissue biopsy.

### 5.2 Demographic, health, and functional measures

#### 5.2.1 Cardiorespiratory fitness (VO_2_ peak)

Cardiorespiratory fitness was measured using gold standard VO_2_ peak (mL/min) from Cardiopulmonary Exercise Testing (CPET) (Cummings et al., 2023). A standardized CPET, using a modified Balke or manual protocol, was administered to participants to measure ventilatory gases, oxygen, and carbon dioxide inhaled and exhaled during exercise. Two slow 5‐min walking tests were conducted before and after the maximal effort test to assess walking energetics at preferred walking speed and a slow fixed speed of 1.5 mph. Participants who were excluded from the maximal effort symptom‐limited peak test had acute electrocardiogram (ECG) abnormalities, uncontrolled blood pressure, or history of myocardial infarction, unstable angina or angioplasty in the preceding 6 months. Testing for VO_2_ peak began at the participant's preferred walking speed with incremental rate (0.5 mph) and/or slope (2.5%) increased in 2‐min stages until respiratory exchange ratio, ratio between VCO_2_ and VO_2_, was ≥1.05 and self‐reported Borg Rating of Perceived Exertion was ≥17. Blood pressure, pulse oximetry, and ECG were monitored throughout exercise. The VO_2_ peak was determined in the BREEZESUITE software (MGC Diagnostics, St. Paul, MN) as the highest 30‐s average of VO_2_ (L/min) achieved. The data were manually reviewed to ensure the correct VO_2_ peak was selected for each participant.

#### 5.2.2 Other measures

Participants were asked to walk at their usual pace for 400 m from which walking speed (m/s) was calculated (Cummings et al., 2023). Whole‐body D_3_Cr muscle mass was measured in participants using a d3‐creatine dilution protocol as previously described. Knee extensor leg power was assessed using a Keiser Air 420 exercise machine in the same leg as the muscle biopsy. Resistance to test power was based on determination of the 1‐repetition maximum leg extensor strength. Weight was assessed by balance beam or digital scales, and height by wall‐mounted stadiometers. An approximately 6‐min‐long MR scan was taken of the whole body to assess body composition including thigh muscle volume with image processing by AMRA Medical. The CHAMPS questionnaire was used to assess specific types and the context of physical activities. Participants were asked to self‐report physician diagnosis of cancer (excluding nonmelanoma skin cancer), cardiac arrythmia, chronic kidney disease, chronic obstructive pulmonary disease, coronary artery disease, congestive heart failure, depression, diabetes, stroke, and aortic stenosis; from this list, a multimorbidity count (0, 1, or 2+) was calculated.

#### 5.2.3 Body size

Weight was assessed by balance beam or digital scales, and height by wall‐mounted stadiometers.

### 5.3 Gene expression and mitochondrial respiration measurements

#### 5.3.1 Skeletal muscle biopsy collection and processing

Percutaneous biopsies were collected from the middle region of the musculus vastus lateralis under local anesthesia using a Bergstrom canula with suction. Biopsies of the vastus lateralis were obtained between 9 am and 11 am on Day 3 of the baseline visit. Participants were eligible for tissue collection after confirming that they were not currently taking blood thinners, had avoided strenuous physical activity for the prior 48 h, had systolic blood pressure lower than 180 mm Hg and diastolic blood pressure lower than 110 mm Hg, recorded no use of aspirin or anti‐inflammatory medications over the prior 3 days, and confirmation that participant was fasted (at least 8 h). Biopsy samples were obtained using a Bergström trocar (5 or 6 mm) with suction applied using a 60 cc syringe and using a local anesthetic (1 or 2% lidocaine HCL). The specimen was blotted dry of blood and interstitial fluid and dissected free of any connective tissue and intermuscular fat. Approximately 20 mg of the biopsy specimen was placed into ice‐cold BIOPS media (10 mM Ca–EGTA buffer, 0.1 M free calcium, 20 mM imidazole, 20 mM taurine, 50 mM potassium 2‐[N‐morpholino]‐ethanesulfonic acid, 0.5 mM dithiothreitol, 6.56 mM MgCl2, 5.77 mM ATP, and 15 mM phosphocreatine [PCr], pH 7.1) for respirometry, as previously described (Mau et al., 2023). Myofiber bundles of approximately 2–3 mg were teased apart using a pair of sharp tweezers and a small Petri dish containing ice‐cold BIOPS media. After mechanical preparation, myofiber bundles were chemically permeabilized for 30 min with saponin (2 mL of 50 μg/mL saponin in ice‐cold BIOPS solution) placed on ice and a rocker (25 rpm). Myofiber bundles were washed twice (10 min each) with ice‐cold MiR05 media (0.5 mM ethylenediaminetetraacetic acid, 3 mM MgCl2·6H2O, 60 mM K‐lactobionate, 20 mM taurine, 10 mM KH2PO4, 20 mM N‐2‐hydroxyethylpiperazine‐N′‐2‐ethanesulfonic acid, 110 mM sucrose, and 1 g/L bovine serum albumin, pH 7.1) on an orbital shaker (25 rpm). The second wash in MiR05 contained blebbistatin (25 μM), a myosin II ATPase inhibitor that was used to inhibit muscle contraction. Fiber bundle wet weight was determined immediately after permeabilization using an analytical balance (Mettler Toledo, Columbus, OH).

#### 5.3.2 Mitochondrial respiration

Maximal complex I‐ and II‐supported oxidative phosphorylation (P1‐Max OXPHOS, also known as State 3 respiration) was measured in permeabilized muscle fiber bundles from biopsies as previously described (Mau et al., 2023). A standardized substrate uncoupler inhibitor titration (SUIT) protocol was run in duplicate to assess the activity of mitochondrial electron transport system in permeabilized muscle fibers (PMF). Following weighing, the PMF bundles were then transferred to the respiration chambers of an Oxygraph 2 K instrument (Oroboros Inc., Innsbruck, Austria). Assays were run at 37°C in MiR05 supplemented with blebbistatin (25 μM) while O_2_ concentration in respiratory chambers were maintained between 400 and 200 μM. Maximal complex I and II‐supported oxidative phosphorylation (Max OXPHOS, also known as State 3 respiration) was measured in the presence of pyruvate (5 mM), malate (2 mM), glutamate (10 mM), succinate (10 mM), and ADP (4.2 mM). Cytochrome c (10 μM) was used to test the integrity of the outer mitochondrial membrane, and any sample showing a response greater than 15% was omitted from analysis. Steady‐state O_2_ flux data was analyzed and normalized to fiber bundle wet weight using Datlab 7.4 software. Respiratory control ratio (RCR, ADP stimulated respiration/non‐ADP‐stimulated respiration in the presence of pyruvate and malate) was 7.08 ± 3.26. The mean coefficient of variation for duplicates of Max OXPHOS measurement was 11.5% across both clinical sites.

#### 5.3.3 RNA library preparation and sequencing

Total RNA from frozen human skeletal muscle samples (~5–30 mg) was prepared using Trizol solution (Invitrogen) according to manufacturer's direction in 2.0 mL Eppendorf safe‐lock tubes. Homogenization was performed using the Bullet Blender (NextAdvance, Raymertown NY USA) with an appropriate quantity of stainless‐steel beads (autoclaved, 0.5 ~ 2 mm, NextAdvance, Raymertown NY USA) at 4°C on Setting 8 (Max is 12) in 30 s bouts. The homogenization step was repeated five times for a total of 3 min with at least 1 min break between each bout. The removal of residual genomic DNA was performed by incubating the RNA sample with DNase (AM1907, Thermosci) plus RiboLock RNase inhibitor (EO0381, Thermisci) at 37°C for 25 min in a heating block (400 rpm). Cleanup of the RNA samples was done using the DNase inactivation reagent following instructions provided by the manufacturer (AM1907, Thermosci). The RNA concentration and integrity were determined by using ThermoSci Nanodrop and Agilent Tapestation.

To prepare RNAseq library, polyA mRNA was isolated from about 250 ng total RNA by using NEBNext Poly(A) mRNA magnetic isolation module (E7490L, NEB) and mRNA library was constructed by using NEBNext Ultra II directional RNA library Pre Kit for Illumina (E7760L, NEB). Equal molarity of RNAseq libraries were pooled and sequenced on Illumina NovaSeq (2X150bp) to reach 80 M reads per sample.

#### 5.3.4 Alignment and quality control

The reads from RNA‐sequencing were aligned to the Genome Reference Consortium Human Build 38 (GRCh38) using the HISAT2 software. Duplicated aligned reads were further marked and excluded using the Picardtools software (http://broadinstitute.github.io/picard/). Expression count data were obtained using the HTseq software. Genes with a total count of ≤20 across all samples were filtered out to remove non‐expressed genes. The quality of the RNASeq data was gauged by the alignment rate and the duplication rate.

#### 5.3.5 Association analyses

Expression levels of 260 genes related to autophagy regulation (56 genes), selective autophagy of mitochondria (mitophagy) (64 genes), mTOR and upstream pathways (121 genes), or a combination of the three gene sets (19 genes) were analyzed (Bordi et al., 2021). Gene expression associations with traits were identified using negative binomial regression models as implemented by DESeq2 in R and adjusted for age, gender, clinic site, race/ethnicity, height, weight, hours per week in all exercise‐related activities (CHAMPS), multimorbidity count category, and sequencing batch. DESeq2 uses a negative binomial generalized linear model for differential analysis and applies the Wald test for the significance of GLM coefficients. The Benjamini–Hochberg false discovery rate method was used for *p*‐value adjustment. Genes were considered differentially expressed according to the significance criteria of FDR <0.05. In negative binomial models, traits were modeled using the number of standard deviations (SDs) from each trait's mean. Consequently, the reported log‐base 2‐fold changes reflect the change in gene expression per one SD unit increase in each trait. Volcano plots were created to visualize the differential expression of RNAs (ENSGs) associated with functional measures. Heat maps were created to summarize significant ENSG associations across all analyzed traits.

## AUTHOR CONTRIBUTIONS

Peggy M Cawthon, Paul M Coen, Steven R Cummings, Steven B Kritchevsky, Anne B Newman, Russell T Hepple, Karyn Esser and Gregory J Tranah designed the study. Paul M Coen and Gregory J Tranah drafted the manuscript. Xiping Zhang, Kevin Wu and Christopher Wolff conducted the sample preparation. Haley N Barnes, Paul M Coen, Daniel S Evans, and Zhiguang Huo conducted the genetic analyses. Paul M Coen, Steven R Cummings, Steven B Kritchevsky, Anne B Newman, Russell T Hepple, Karyn Esser, Olaya Santiago Fernandez, Ana Maria Cuervo, and Gregory J Tranah contributed to the interpretation of the results. Peggy M Cawthon, Paul M Coen, Frederico GS Toledo, Steven B Kritchevsky, Anne B Newman, Steven R Cummings, and Gregory J Tranah have critically revised the manuscript. All authors reviewed and approved the final version of the manuscript and Haley N Barnes and Zhiguang Huo had full access to the data in the study and accept responsibility to submit for publication.

## FUNDING INFORMATION

SOMMA is funded by the National Institute on Aging (NIA) grant number R01AG059416. Study infrastructure support was funded in part by NIA Claude D. Pepper Older American Independence Centers at University of Pittsburgh (P30AG024827) and Wake Forest University (P30AG021332) and the Clinical and Translational Science Institutes, funded by the National Center for Advancing Translational Science at Wake Forest University (UL10TR001420).

## CONFLICT OF INTEREST STATEMENT

None declared.

## PATIENT CONSENT

All participants provided written informed consent.

## Supporting information


Tables S1–S8.

## Data Availability

All SOMMA data are publicly available via a web portal. Updated datasets are released approximately every 6 months (https://sommaonline.ucsf.edu).
