# Supplementary material for: Autophagy gene expression in skeletal muscle of older individuals is associated with physical performance, muscle volume and mitochondrial function in the study of muscle, mobility and aging (SOMMA)
Source: Aging Cell. 2024 Apr 16;23(6):e14118. doi: 10.1111/acel.14118 (PMC11166359; doi:10.1111/acel.14118)
Supplement: Supplementary file 1 — Tables S1–S8. [file ACEL-23-e14118-s001.pdf]

Supplemental Table 1

Sample	Number of raw pairs	Alignment Rate	Number of aligned reads	Duplication Rate	Number of aligned reads deduplicated
1	96393453	95.67%	184441951	78.70%	39359245
2	100059418	96.13%	192367958	68.10%	61433169
3	93532566	96.08%	179727375	68.50%	56555649
4	90130020	95.93%	172917121	62.90%	64215917
5	93513176	95.74%	179061926	66.50%	60023883
6	93399507	96.03%	179390873	78.60%	38357663
7	91533891	96.04%	175814274	75.50%	43079694
8	95660352	95.15%	182046440	86%	25426123
9	92869196	95.60%	177559973	73.80%	46546318
10	94206508	95.61%	180140177	65.40%	62368154
11	91580510	96.24%	176269851	68.30%	55963786
12	96476311	96.31%	185835269	65.40%	64275793
13	93256753	96.06%	179170646	69.90%	53926180
14	94737694	96.51%	182871302	70.80%	53335997
15	94853873	94.61%	179484404	91.60%	15094298
16	93254346	96.10%	179236781	79.80%	36214863
17	94768451	96.06%	182078220	70.30%	53994168
18	92703785	96.36%	178650402	61.20%	69327789
19	92792742	96.33%	178781101	65.80%	61190811
20	95448973	96.50%	184214920	71.60%	52242426
21	96386549	95.70%	184490763	65.20%	64208390
22	94049712	95.77%	180144045	85.40%	26374250
23	92534259	95.52%	176781852	82%	31751068
24	96136369	95.47%	183567705	77.70%	40894545
25	92684795	96.66%	179169760	79%	37553138
26	94274385	95.62%	180291886	81%	34329087
27	95238852	95.32%	181565967	81.80%	32954237
28	94658476	95.26%	180343006	89.90%	18235077
29	92917699	95.43%	177341522	75.20%	43975639
30	97826542	96.21%	188243115	72.50%	51785757
31	93211235	95.45%	177942884	83.40%	29533846
32	95606463	95.69%	182973610	75.20%	45433035
33	95441961	96.31%	183849801	74.60%	46701458
34	96075319	96.12%	184697195	74.20%	47673981
35	95074060	96.35%	183208134	70.80%	53417857
36	91693188	95.82%	175727627	77.60%	39393394
37	92617017	92.67%	171647448	90.10%	16974404
38	95760744	95.74%	183363658	78%	40363306
39	93959617	94.95%	178419945	82.10%	32008520
40	97687230	95.69%	186945742	72.80%	50891281
41	95290985	96.08%	183118608	76.10%	43755133
42	96405400	96.10%	185288591	71.30%	53153408
43	91618248	95.70%	175361499	74%	45638953
44	90604635	95.59%	173221766	79.70%	35235993
45	91908525	94.24%	173221121	73.90%	45147052
46	93339668	95.45%	178183219	86.60%	23847280
47	93228195	95.38%	177838787	80.10%	35379447
48	94185936	95.70%	180277596	85.90%	25478974
49	93076777	95.60%	177965955	74.90%	44623024
50	92853231	95.51%	177372625	67.50%	57638727
51	95924802	95.83%	183844971	83.20%	30890475
52	91701902	93.61%	171676642	88.50%	19794352
53	93978044	95.60%	179680494	82.20%	31952553
54	93498088	94.84%	177339169	85.90%	25052790
55	91373701	95.10%	173792287	86%	24339052
56	92633161	94.21%	174537705	92.40%	13282764
57	93632029	94.82%	177555337	74.70%	44895907
58	96935810	96.11%	186322556	66.40%	62655363
59	95970326	95.97%	184214134	68.70%	57608266
60	92800167	95.46%	177179393	86.60%	23666468
61	102699034	96.30%	197801041	64.90%	69504546
62	92654946	94.91%	175870882	85%	26296527
63	92637261	94.25%	174620099	85.40%	25547736
64	93933534	94.91%	178301144	86%	24893302
65	94850969	94.69%	179622664	91.10%	15967433
66	93576231	95.41%	178562087	80.90%	34065450
67	90485671	95.26%	172384541	75.90%	41587116
68	93379297	94.19%	175909909	91.20%	15397217
69	96498988	95.91%	185101771	73.10%	49753846
70	71461019	95.40%	136344256	59.30%	55504737
71	93752448	95.60%	179248936	59.20%	73073568
72	86411126	96.23%	166306753	58.30%	69376925
73	83235259	96.26%	160250568	55.50%	71262583
74	81261596	96.36%	156615091	60.10%	62471112
75	97783727	95.85%	187450816	57.10%	80348879
76	83767720	95.75%	160407526	57.60%	68092398
77	86729806	95.31%	165318863	57.60%	70036456
78	110942864	95.51%	211919823	57%	91162871
79	104966827	96.19%	201945250	56%	88772846
80	98253075	96.48%	189598150	65.60%	65198970

81	116927639	96.27%	225129926	56.50%	97959452
82	112176949	96.19%	215797160	58.40%	89813138
83	98534039	95.80%	188796585	53.50%	87847469
84	108954720	95.86%	208892196	53.40%	97416327
85	84097255	95.78%	161090321	57.10%	69129756
86	95236027	95.90%	182663085	61.60%	70206810
87	88597819	95.87%	169884224	55.30%	75955704
88	96328455	96.12%	185180033	57.80%	78177200
89	90461109	96.09%	173855662	56.80%	75088574
90	102870253	95.60%	196695477	57.10%	84377364
91	108655599	96.07%	208762677	55%	93925156
92	106334623	95.39%	202873100	58%	85169516
93	95273543	96.52%	183917484	59.50%	74534598
94	92910303	95.10%	176722146	61.70%	67734224
95	85877520	95.74%	164431755	57.30%	70181106
96	101420521	96.01%	194746668	57.80%	82270980
97	106560498	95.79%	204151071	60.10%	81465013
98	88052217	95.85%	168790927	61.40%	65182021
99	86639281	95.98%	166305148	60.20%	66160365
100	110166267	96.12%	211781602	58.40%	88101366
101	68517291	95.90%	131415364	58.40%	54667509
102	82185872	89.49%	147099825	56.90%	63463056
103	81881951	91.49%	149826924	51.60%	72577249
104	79982310	92.80%	148454721	54.20%	68030223
105	80404905	93.43%	150237431	54.10%	68971717
106	80821283	92.89%	150151720	56.80%	64805299
107	81203942	92.14%	149636180	52.50%	71064306
108	83124572	93.65%	155692925	55%	70035174
109	83732758	91.81%	153750133	56%	67697013
110	85425657	93.21%	159252490	55.20%	71368045
111	83608132	92.59%	154821249	55.10%	69477695
112	80057039	92.61%	148280864	51.40%	72014599
113	80421460	92.78%	149231205	55.70%	66145867
114	79661371	93.39%	148792790	52.40%	70837393
115	78552545	93.67%	147161472	49.60%	74116342
116	80742166	93.70%	151310619	53.80%	69932072
117	81952954	93.45%	153170108	51.40%	74467136
118	84605721	92.48%	156488222	53.50%	72754366
119	83317876	93.43%	155693857	54.60%	70715421
120	84072070	92.31%	155213840	55.10%	69658730
121	78879445	93.85%	148060431	54.30%	67613149
122	81065245	93.13%	150987465	52.90%	71065006
123	80217494	93.74%	150386891	52.60%	71283062
124	78825058	93.19%	146907604	53%	69007099
125	82173370	92.22%	151561681	56%	66722801
126	84325245	91.89%	154967259	55.80%	68529272
127	83005575	92.12%	152925352	52.40%	72862136
128	79764300	92.28%	147216468	54.60%	66881432
129	80033284	93.50%	149658311	53.60%	69425984
130	83094845	92.07%	153013625	56%	67339202
131	81833515	93.24%	152604746	53.20%	71429012
132	81767329	92.75%	151672136	54.50%	69022261
133	86613650	93.83%	162536626	50.90%	79868855
134	84575964	93.04%	157374691	52.80%	74277949
135	84551330	93.20%	157608706	57.30%	67364855
136	79377942	92.20%	146379254	54.60%	66496956
137	82220781	93.55%	153841128	53.80%	71087389
138	84490367	93.66%	158262891	49%	80737589
139	79135730	93.46%	147925972	54.60%	67200337
140	79315800	93.29%	147985482	53.80%	68321650
141	83183251	93.78%	156011569	56.20%	68318618
142	80033861	93.20%	149184350	54.50%	67886689
143	81365287	93.54%	152211890	53.10%	71334270
144	78302039	93.65%	146653385	52%	70451521
145	78250641	93.72%	146677528	50.20%	72978169
146	88982141	93.56%	166509772	51.30%	81144529
147	83465583	93.76%	156512508	52.70%	73991949
148	78062842	93.16%	145453918	54.90%	65605230
149	82988051	93.61%	155366184	54.10%	71328165
150	81862895	93.18%	152566757	53.50%	70874912
151	83357795	92.99%	155033190	53.90%	71547631
152	78541474	92.61%	145477010	55.20%	65238613
153	79658492	93.21%	148502581	54%	68314118
154	83568623	93.12%	155630779	51.10%	76070555
155	82216893	93.36%	153507981	54.80%	69417332
156	80284841	93.01%	149342427	53.80%	69032431
157	81855521	92.63%	151649262	57.70%	64177810
158	83777511	92.15%	154404604	56.40%	67384639
159	76397124	92.84%	141852133	52.10%	67974888
160	76699266	93.64%	143635884	52.50%	68190049
161	81119697	92.98%	150848135	57.30%	64381281
162	80828343	93.88%	151762556	53.70%	70201572
163	81754170	92.54%	151315363	54.40%	68934509

164	77182270	92.88%	143378696	54.50%	65188366
165	83277951	91.93%	153115251	54.80%	69242593
166	81434647	93.24%	151858802	53%	71313059
167	80097377	93.07%	149095520	56%	65587909
168	80351222	92.75%	149049068	55.40%	66477867
169	81065470	92.60%	150136734	56.50%	65376792
170	79732292	92.77%	147932802	51.20%	72190979
171	81364923	92.82%	151041094	54.70%	68468523
172	84217555	92.71%	156156243	53.40%	72719070
173	82659700	91.86%	151855951	58.90%	62427158
174	77764144	92.67%	144122794	54.70%	65326768
175	78458276	93.61%	146894807	53.40%	68434532
176	82281245	92.59%	152375583	54.70%	69096970
177	81723750	93.04%	152071478	53.70%	70467804
178	92966002	93.08%	173061954	53%	81409313
179	108642167	92.90%	201857878	54.50%	91931884
180	110042963	93.11%	204915230	55.40%	91326091
181	80782419	91.74%	148214005	55.40%	66133720
182	84525342	93.26%	157656520	54.20%	72184934
183	83707351	92.93%	155580510	56.70%	67374897
184	81396801	92.67%	150863483	54.20%	69072120
185	79461855	92.93%	147683370	53.60%	68516893
186	82835786	93.45%	154824888	56.10%	68011860
187	80121046	91.52%	146653254	56.20%	64198018
188	80677720	93.08%	150191167	51.70%	72467988
189	73976840	92.95%	137522860	55.50%	61139754
190	84296590	92.68%	156244470	54.30%	71397717
191	79357053	92.05%	146094379	57.40%	62260719
192	83738743	92.78%	155381196	55.10%	69733453
193	81718359	93.00%	151991385	55.70%	67276304
194	82041039	92.73%	152145911	53.70%	70411580
195	80029302	93.27%	149286808	54.60%	67736131
196	80785074	92.28%	149102807	58.20%	62289713
197	83843652	89.95%	150834458	55.60%	66959638
198	83590981	90.54%	151374766	54.40%	69016209
199	83437723	90.56%	151119766	61.30%	58460828
200	83200552	90.82%	151130394	58.30%	63046406
201	85626471	91.26%	156284569	54.70%	70851462
202	85639782	91.15%	156127532	55.30%	69759580
203	82537377	92.16%	152128868	54.80%	68808968
204	85570850	91.07%	155854052	55.50%	69431392
205	85934669	88.37%	151874286	57.70%	64291224
206	82777122	89.44%	148070369	56.30%	64655527
207	84965709	91.77%	155946997	60.90%	60917009
208	83933360	91.66%	153860969	56.30%	67229061
209	84894553	91.66%	155625165	54.60%	70729417
210	86259859	91.23%	157396509	57.40%	67053274
211	84346300	90.21%	152170674	60.30%	60352643
212	81677653	92.44%	151003784	54%	69511530
213	84048781	90.99%	152954587	54.50%	69606002
214	83654135	91.72%	153456971	56.10%	67299283
215	82130420	91.59%	150453661	55.80%	66435733
216	86286063	92.62%	159833996	55.20%	71548012
217	81626143	92.37%	150800166	53.50%	70150436
218	88346127	92.33%	163143773	61.10%	63421969
219	84865918	91.48%	155278413	59.90%	62191416
220	84973197	91.01%	154664003	57.10%	66420352
221	85254795	91.64%	156259490	52.70%	73942216
222	83789654	91.01%	152511134	58.80%	62864169
223	95815599	92.47%	177201844	57.80%	74694621
224	81589169	92.29%	150602813	53.40%	70253955
225	84008407	93.45%	157012490	54.40%	71645670
226	91458190	92.84%	169827777	52%	81521417
227	85816384	92.18%	158208155	51.90%	76103627
228	85124387	92.51%	157502606	53.30%	73491302
229	95718199	92.66%	177393846	50.90%	87012401
230	88765558	91.84%	163040208	50.20%	81131345
231	85307518	92.10%	157139563	47.20%	82950928
232	85215525	92.17%	157078878	52.20%	75159281
233	92867670	92.78%	172320565	50.30%	85711818
234	95802997	92.10%	176464214	51%	86392608
235	90056750	92.42%	166462816	52.40%	79208945
236	88674254	92.26%	163617746	49.10%	83319295
237	87963713	93.34%	164213747	49.40%	83028930
238	92809866	92.70%	172061848	51.70%	83186393
239	87668758	92.36%	161935944	54.80%	73257571
240	83691132	93.13%	155876722	52.30%	74285916
241	87620635	93.43%	163722214	48.50%	84303328
242	87370476	93.83%	163955893	51.50%	79457633
243	85183296	93.51%	159304394	48.90%	81388632
244	85305143	91.00%	155259108	59.40%	62964001
245	87686269	91.76%	160913835	50.10%	80229213
246	85759147	92.88%	159301455	45.80%	86380616

247	86761827	92.46%	160444212	52.30%	76597750
248	95574789	93.14%	178043997	53.60%	82642295
249	84799240	91.03%	154390548	58.90%	63417450
250	92994203	92.25%	171580708	54.90%	77303002
251	90002275	93.51%	168319214	52.50%	79979804
252	93496332	92.41%	172791290	53.20%	80942510
253	93429096	93.13%	174026090	53.40%	81155282
254	92210444	93.87%	173118774	49.60%	87229532
255	86830309	93.01%	161524479	51.20%	78790992
256	85668489	93.69%	160529297	53.20%	75101647
257	82811822	93.50%	154851807	56.70%	67098167
258	92210607	92.75%	171043151	56.20%	74920206
259	86670973	92.98%	161175596	54%	74204742
260	85317863	92.59%	157985436	53.60%	73349237
261	84376356	93.29%	157430753	52%	75551090
262	88846406	93.69%	166474759	52.80%	78535808
263	84250864	93.37%	157323970	49.90%	78810698
264	83884503	93.41%	156707758	55.70%	69362822
265	84039086	93.25%	156727952	58.10%	65613249
266	85755986	93.72%	160745223	52.40%	76444502
267	84323917	93.02%	156873725	55.10%	70465299
268	86565553	93.53%	161926567	50.80%	79604802
269	86567025	93.16%	161289477	50.10%	80523325
270	88494339	92.87%	164361568	52%	78929966
271	86623009	93.02%	161151152	51.20%	78600588
272	86327648	93.10%	160745980	54%	73918147
273	83141346	93.24%	155036034	53.30%	72386235
274	83302444	93.24%	155343331	60.30%	61680278
275	84909067	93.47%	158736296	53.10%	74517144
276	94998934	93.04%	176782669	54.20%	80973713
277	85473106	91.72%	156783969	48%	81529587
278	84419283	93.08%	157151408	49.60%	79147797
279	84339155	92.54%	156093345	54.10%	71659275
280	82898656	93.49%	155005772	57.10%	66469529
281	88618338	92.36%	163687680	55.50%	72864712
282	84028068	92.83%	155998753	55.40%	69596791
283	86880065	92.54%	160795831	54.20%	73623117
284	84845899	93.53%	158711551	51.20%	77485314
285	84860629	92.11%	156330197	52.40%	74379075
286	84257108	93.42%	157421881	52.40%	74920353
287	84280613	93.66%	157878588	50.10%	78725756
288	84861735	93.52%	158721939	53.70%	73496627
289	85386584	93.20%	159158664	51.50%	77183783
290	85547278	91.97%	157357004	64.40%	56086510
291	85506856	92.17%	157623630	50.60%	77883014
292	87465435	92.87%	162465151	48.70%	83288893
293	89476865	93.44%	167220449	50.30%	83141110
294	82110594	93.53%	153591414	55%	69078281
295	84093778	92.59%	155729679	60.40%	61678538
296	83423489	93.01%	155185118	59.60%	62749001
297	85932540	93.29%	160337553	59.40%	65131305
298	83538416	93.34%	155953809	52.40%	74289150
299	84091188	93.52%	157277665	49.20%	79941873
300	90069946	93.01%	167540972	52.10%	80254592
301	84061111	93.66%	157457891	50.80%	77394403
302	83155272	93.16%	154932286	57.50%	65789921
303	83585064	92.55%	154711276	56.30%	67639225
304	84628574	93.46%	158188844	55.90%	69742868
305	83011782	92.14%	152977493	54.20%	70059215
306	81280441	93.78%	152451484	50.80%	74998130
307	84245261	92.76%	156299058	54%	71892451
308	84725802	92.59%	156893654	51.20%	76591677
309	84866329	92.66%	157282114	52.60%	74575212
310	85688224	92.84%	159099875	53.30%	74358069
311	85437828	91.87%	156986962	62.70%	58478149
312	84433153	92.86%	156816220	55.70%	69523283
313	84621049	92.92%	157251935	53.20%	73551058
314	83144014	93.91%	156162315	50.30%	77646238
315	83362769	92.86%	154822328	50.70%	76272766
316	84481479	93.31%	157655895	49.30%	79968258
317	86230336	93.73%	161655502	50.80%	79536622
318	84799431	92.73%	157261019	49.80%	78957526
319	78405835	93.18%	146109354	48%	75928263
320	88892787	93.76%	166692480	50.80%	82020430
321	85070862	93.32%	158769321	48%	82521937
322	84947408	94.30%	160214849	50.80%	78829492
323	92407695	93.73%	173228551	52.50%	82368708
324	84891874	94.27%	160048590	50.70%	78926705
325	85709910	94.39%	161804370	51.50%	78417315
326	83101247	94.01%	156241366	48.50%	80410232
327	82308793	93.72%	154274395	52.10%	73843575
328	87109225	94.00%	163759092	51%	80301894
329	86532923	92.20%	159572561	50.90%	78415267

330	90737882	90.54%	164315402	54.90%	74053890
331	83355319	92.29%	153862208	50.30%	76523033
332	85026830	93.60%	159169119	51.60%	76985647
333	83133743	92.89%	154448461	52.50%	73314153
334	81974796	92.52%	151679531	49.70%	76328624
335	85411349	93.16%	159131845	48.70%	81630732
336	87230169	89.93%	156896035	51.20%	76597809
337	86522843	93.28%	161421146	52.10%	77300193
338	83912636	92.91%	155918473	47.40%	81939343
339	96064117	93.21%	179087734	55.50%	79780353
340	91874480	92.19%	169398960	54.10%	77734137
341	90458184	93.45%	169069924	54.20%	77368105
342	92262874	92.32%	170355177	56.30%	74361329
343	88623713	93.33%	165428691	54.40%	75387321
344	87207805	91.69%	159918728	62.10%	60610291
345	91004676	91.07%	165761485	65.50%	57159741
346	90916275	93.80%	170560323	54.10%	78288436
347	82776921	91.55%	151571920	65.70%	52028736
348	92768155	91.99%	170681073	52.80%	80587319
349	87856604	91.93%	161536091	56%	71121274
350	94246257	93.71%	176642932	54%	81343271
351	90609020	92.28%	167228668	60.30%	66404884
352	96498298	92.76%	179016244	60%	71599729
353	90315456	92.62%	167293252	57.70%	70834030
354	91413142	91.00%	166373604	59.60%	67260982
355	87915585	92.42%	162503027	61.70%	62305118
356	91125683	92.91%	169336075	57.40%	72191861
357	91398473	92.76%	169571520	54.20%	77661393
358	95481994	91.81%	175329119	60.40%	69382289
359	110818214	92.60%	205241457	57.60%	87117111
360	94806068	92.95%	176246636	54.70%	79789029
361	89836893	92.71%	166580194	56.10%	73142972
362	98638711	93.23%	183921845	56.70%	79659069
363	93853377	92.12%	172917042	55.50%	77019448
364	100275484	91.79%	184078267	58.40%	76604986
365	92495429	93.01%	172062371	54.80%	77718704
366	88715899	92.84%	164731494	55.50%	73260860
367	92461693	93.31%	172556715	55.80%	76329898
368	102100512	92.74%	189382528	54%	87061978
369	90504044	92.70%	167798855	60.10%	66899554
370	89709289	93.94%	168552896	52.20%	80537509
371	87126691	93.41%	162764208	60.10%	64993578
372	84451594	93.81%	158447618	54.30%	72379951
373	92484999	93.30%	172582788	56.20%	75514636
374	95069991	92.37%	175627025	53.60%	81522149
375	91417795	93.66%	171251489	54.30%	78191020
376	87960991	90.90%	159916106	59.70%	64483465
377	99886475	93.44%	186660828	57.40%	79514036
378	87890978	92.47%	162553511	57.30%	69367802
379	92017579	93.07%	171275765	61.20%	66437963
380	87881414	93.31%	163996843	54.10%	75296785
381	88237604	93.83%	165581376	52.10%	79392676
382	87808127	93.01%	163340130	55.70%	72401527
383	91892021	94.02%	172792672	52.20%	82574586
384	86824993	93.68%	162683119	53.70%	75385841
385	83169708	92.44%	153768393	64.50%	54605416
386	92957654	91.89%	170829216	61.60%	65562022
387	90627760	93.92%	170231373	63.60%	61998824
388	94159915	93.71%	176478389	53.60%	81955000
389	93895734	92.31%	173357587	67%	57291911
390	93110943	91.98%	171288798	65.50%	59067248
391	91179562	93.39%	170307295	60.70%	66994666
392	94007538	94.13%	176984606	57%	76037137
393	92783466	91.90%	170532129	65.30%	59175784
394	93347423	93.30%	174193583	54.10%	80031876
395	93719900	92.07%	172581611	56.80%	74499816
396	94289838	91.92%	173338731	68.30%	54872025
397	94653775	93.44%	176883243	62.80%	65720019
398	94164370	93.32%	175752605	61.90%	66968050
399	88567436	93.30%	165258210	69.10%	51033967
400	98416526	92.21%	181506660	61%	70857795
401	95476259	92.08%	175822231	74%	45652965
402	92721577	93.59%	173558028	66.90%	57505064
403	92259150	91.10%	168094982	66.80%	55805579
404	88083362	93.76%	165177022	58.10%	69219073
405	92673640	92.23%	170944821	68.20%	54331377
406	91470878	90.71%	165950945	68.30%	52630329
407	92289829	91.24%	168411040	66.50%	56472297
408	88452199	92.23%	163152205	63.10%	60181547
409	94457069	91.78%	173385603	69.20%	53418541
410	90804922	90.60%	164542909	59.40%	66759518
411	92170656	92.31%	170159210	58.80%	70080399
412	88015234	90.63%	159541716	67.40%	52066946

413	90896266	92.02%	167291419	66.50%	55964132
414	95267470	93.90%	178918725	63.10%	66019900
415	90307683	89.91%	162395740	75.20%	40311703
416	89954030	93.51%	168237445	72.70%	45963463
417	92393129	90.77%	167722925	58.60%	69448174
418	96504239	92.38%	178305273	54.50%	81161684
419	94181054	92.24%	173736160	53.60%	80633299
420	88132332	92.81%	163596957	55.20%	73288089
421	92289521	93.42%	172434278	56.30%	75417880
422	93722558	91.93%	172308930	59.10%	70468747
423	91533009	90.67%	165985429	72.30%	45947853
424	98072046	92.20%	180847605	66.80%	59997635
425	94242577	88.97%	167693401	61.30%	64908370
426	90280373	91.21%	164689198	66.60%	54965722
427	93086902	94.13%	175245153	62.80%	65237940
428	88128911	92.82%	163601591	63.40%	59947062
429	93573424	93.78%	175506311	62.50%	65822250
430	93868423	93.42%	175381316	57.60%	74397340
431	98482895	90.88%	179010692	73.80%	46966176
432	93638980	93.02%	174207479	64.10%	62462076
433	89719416	93.27%	167369730	65.70%	57463722
434	95073085	91.95%	174833170	61.70%	66880236
435	89822530	92.97%	167011901	67.30%	54612076
436	91834032	92.67%	170198511	61.20%	66085430
437	92515385	89.52%	165636384	71.80%	46708002
438	81638563	93.65%	152909707	55.20%	68573636
439	100452737	93.09%	187024703	54.60%	84975617
440	96805206	92.74%	179556773	65.90%	61168508
441	98930632	93.51%	185023729	58.70%	76505368
442	96017904	92.77%	178147884	58.20%	74503587
443	90936815	93.73%	170469363	58.90%	70128609
444	89671949	92.11%	165189706	63.80%	59734038
445	93655808	93.56%	175242283	56.20%	76800194
446	92481706	93.39%	172736906	60.80%	67797725
447	92242548	93.29%	172115150	58.20%	71974570
448	98958108	92.46%	183000445	54.90%	82605208
449	89329760	93.54%	167121757	57.40%	71144290
450	92546113	92.53%	171258949	52.70%	80939003
451	95495914	90.44%	172734328	64.70%	60986570
452	90617866	91.86%	166476607	56.70%	72097969
453	85249239	92.77%	158175937	50%	79019520
454	96473089	93.21%	179838851	61.30%	69667130
455	87622388	93.39%	163658186	52.40%	77843675
456	106753538	95.40%	203694475	53.80%	94184243
457	97876344	94.93%	185836410	59.10%	75983609
458	96724752	94.96%	183708439	56.70%	79591673
459	98803703	94.37%	186480153	56.10%	81804328
460	93016321	94.98%	176700272	54%	81212759
461	92084819	94.83%	174656278	55.80%	77212005
462	95001560	93.67%	177982126	57.20%	76218237
463	106809646	94.65%	202187866	57.80%	85413716
464	87839255	94.12%	165355226	52.80%	77967579
465	105811413	94.38%	199739189	56.70%	86405329
466	95541031	91.59%	175007190	69.10%	54148777
467	100430849	94.49%	189789449	58.90%	77935513
468	105982368	93.82%	198873360	56.20%	87132432
469	101514895	94.94%	192751591	50.30%	95763249
470	94660040	89.61%	169654972	59.10%	69446557
471	101901254	95.58%	194798665	54.90%	87802945
472	94699721	94.57%	179109617	57.20%	76588848
473	105237557	94.78%	199487211	54.10%	91466671
474	95567120	96.24%	183956252	62.70%	68536981
475	94395254	96.17%	181562730	58.70%	74947279
476	98761952	94.66%	186975979	58.10%	78341010
477	92467802	94.89%	175485793	59.10%	71696131
478	96982315	94.40%	183094897	61.40%	70687779
479	101831651	94.93%	193331574	53.20%	90575634
480	97300773	95.01%	184887565	52.60%	87728858
481	96666603	93.43%	180640088	63.40%	66139023
482	102251855	95.72%	195760752	58.30%	81682880
483	97637176	94.63%	184781648	52.90%	86991385
484	101283273	93.99%	190390008	57.20%	81456991
485	101091302	94.91%	191881678	57.90%	80701975
486	93013111	94.11%	175065722	59.80%	70354519
487	105604071	94.43%	199445496	54.20%	91328980
488	93988400	93.25%	175281809	64.30%	62488580
489	99689678	94.93%	189267116	59.30%	77096563
490	103505998	95.05%	196756837	58.80%	80978414
491	97155437	94.88%	184359341	61%	71943927
492	97162311	94.64%	183899317	64.20%	65825221
493	97734107	94.45%	184624265	62.60%	69118740
494	97047216	94.80%	183998443	63.20%	67785009
495	97440239	94.48%	184125148	54.40%	84029016

496	94981444	94.62%	179740485	62.30%	67796232
497	90898018	93.98%	170847926	62.10%	64797984
498	98192837	95.43%	187415265	57.50%	79655358
499	94635900	95.09%	179972994	57%	77352751
500	96304744	95.18%	183331357	58.10%	76877869
501	99152855	94.85%	188094606	52.80%	88830603
502	87912768	94.13%	165498166	57.90%	69747262
503	102072863	94.93%	193786918	58.80%	79862675
504	95626828	94.70%	181125533	62.10%	68618652
505	95462159	95.32%	181984022	58.90%	74786308
506	86963075	94.99%	165212291	59.70%	66591725
507	102034874	94.81%	193475179	62.70%	72089086
508	93891414	94.86%	178132723	61.10%	69241995
509	95377432	94.81%	180862233	61.40%	69852609
510	105657361	94.56%	199815540	59.40%	81070642
511	94505366	95.15%	179845898	54.60%	81647583
512	93503827	95.19%	178005941	92.80%	12853785
513	97584136	94.64%	184705043	55.40%	82434795
514	94742267	94.40%	178876936	57.80%	75464728
515	96662924	95.12%	183899328	60.60%	72487446
516	95153939	94.63%	180092578	60.40%	71351472
517	94091898	93.90%	176704699	62.10%	66893054
518	92269025	94.15%	173743946	59.40%	70526796
519	94381366	94.39%	178175304	56.20%	77967554
520	95654141	95.06%	181862423	58%	76350924
521	97093342	94.96%	184396026	58.60%	76254633
522	92388585	95.13%	175771350	58.80%	72358164
523	92060426	94.69%	174350333	58%	73313254
524	109881129	94.29%	207219855	57.20%	88731020
525	101050070	95.68%	193365348	59%	79184265
526	96318962	94.25%	181563940	58.90%	74608502
527	91688583	94.76%	173766923	58.80%	71569344
528	98328743	95.19%	187205952	58.30%	78136105
529	121446366	94.15%	228685610	57.70%	96642845
530	100138016	94.31%	188880646	61.80%	72140808
531	94415088	95.10%	179586762	57.30%	76701379
532	102141157	95.18%	194426179	55.30%	86963987
533	97730240	95.01%	185713182	54.70%	84165544
534	96131064	95.57%	183745808	55.20%	82243616
535	95363612	94.02%	179323841	59%	73469130
536	102903390	94.13%	193719440	61.20%	75199767
537	100346986	92.46%	185567738	62.10%	70414077
538	100722120	94.73%	190831813	57.70%	80788804
539	95881211	94.30%	180836733	60.70%	71070593
540	95567678	95.28%	182111290	52.60%	86301224
541	97359400	95.05%	185084691	59.30%	75373328
542	97578199	94.91%	185213389	61.30%	71606892
543	96910187	95.58%	185246359	53.70%	85782395
544	112780954	95.18%	214694984	52.20%	102724183
545	93275663	95.35%	177880631	56.20%	77840263
546	83354267	94.64%	157767241	50.80%	77638649
547	90439586	95.09%	171994479	59.60%	69448972
548	103168200	93.69%	193320598	52.20%	92492657
549	98614058	95.08%	187530774	52.90%	88392266
550	98772349	94.43%	186545174	51.80%	89907393
551	96029438	91.99%	176675593	58.20%	73793839
552	94987817	94.13%	178823254	55.70%	79172902
553	98177356	95.20%	186920472	52.80%	88148676
554	94374784	94.06%	177536940	56.20%	77690572
555	99184295	94.78%	188008498	61.80%	71824464
556	96475347	93.23%	179896780	56.40%	78377785
557	95325137	94.82%	180772579	55.80%	79913858
558	99134429	96.11%	190559341	62.80%	70917976
559	95543580	94.65%	180869530	57.70%	76457595
560	101595656	95.06%	193147151	55.40%	86101711
561	91435512	94.70%	173172445	55%	77845967
562	95844954	93.06%	178382933	60%	71414095
563	93400224	94.81%	177107821	55.50%	78810835
564	97693345	93.78%	183240742	55.80%	81060064
565	111457841	94.01%	209556407	54.40%	95599806
566	100924314	94.69%	191128298	60.80%	74961152
567	95146106	94.99%	180753766	60%	72328773
568	92308177	94.52%	174506357	60.40%	69065020
569	93055956	94.71%	176261034	53.60%	81708334
570	94736443	94.54%	179120753	59.50%	72515103
571	101645086	94.82%	192751968	59.50%	78029324
572	95245554	93.82%	178711513	58.70%	73752412
573	94462242	94.09%	177753492	58.60%	73591688
574	94920698	94.50%	179406360	57.90%	75451736
575	93789701	95.31%	178776881	57.80%	75530134

Supplementary Table 2. Gene expression levels stratified by 400m walk speed tertiles

ensembl_gene_id	hgnc_symbc	pathways	TOTALN	OVERALL	Walk Speed Tertiles			PVAL	PTREND
					Tertile 1	Tertile 2	Tertile 3		
					0.458 <= T1 < 1.012	1.012 <= T2 < 1.154	1.154 <= T3 < 1.591		
				(N= 575)	(N= 191)	(N= 192)	(N= 192)		
ENSG00000116016	EPAS1	Autophagy regulators	575	3221.86 +/- 1188.73	2993.50 +/- 1141.40	3303.45 +/- 1262.35	3367.44 +/- 1130.05	0.0043	0.0020
ENSG00000170345	FOS	Autophagy regulators	575	646.12 +/- 1364.77	808.47 +/- 1860.02	570.11 +/- 956.91	560.62 +/- 1095.83	0.1319	0.0758
ENSG00000173039	RELA	Autophagy regulators	575	1064.32 +/- 360.48	1083.92 +/- 387.85	1081.19 +/- 372.14	1027.95 +/- 316.83	0.2304	0.1285
ENSG00000109819	PPARGC1A	Autophagy regulators	575	1697.55 +/- 719.98	1622.14 +/- 667.81	1702.88 +/- 877.44	1767.25 +/- 577.64	0.1419	0.0485
ENSG00000109320	NFKB1	Autophagy regulators	575	736.74 +/- 233.84	770.61 +/- 264.99	736.67 +/- 240.19	703.11 +/- 185.77	0.0183	0.0046
ENSG00000186951	PPARA	Autophagy regulators	575	3072.41 +/- 1257.77	2842.09 +/- 1310.76	3144.07 +/- 1284.68	3229.87 +/- 1145.25	0.0064	0.0025
ENSG00000120738	EGR1	Autophagy regulators	575	488.63 +/- 719.13	525.16 +/- 849.66	502.86 +/- 688.47	438.06 +/- 598.27	0.4690	0.2361
ENSG00000141510	TP53	Autophagy regulators	575	227.87 +/- 79.28	235.57 +/- 86.82	237.22 +/- 83.74	210.86 +/- 62.75	0.0012	0.0022
ENSG00000136997	MYC	Autophagy regulators	575	186.75 +/- 114.61	208.84 +/- 125.64	183.21 +/- 117.74	168.32 +/- 95.25	0.0021	0.0005
ENSG00000131759	RARA	Autophagy regulators	575	391.32 +/- 156.52	394.04 +/- 161.27	403.29 +/- 168.49	376.65 +/- 137.83	0.2387	0.2765
ENSG00000132170	PPARG	Autophagy regulators	575	97.94 +/- 55.90	99.26 +/- 61.47	100.11 +/- 59.96	94.44 +/- 44.91	0.5640	0.3993
ENSG00000104856	RELB	Autophagy regulators	575	120.65 +/- 56.91	127.58 +/- 61.65	125.54 +/- 59.24	108.86 +/- 47.23	0.0019	0.0012
ENSG00000169136	ATF5	Autophagy regulators	575	489.34 +/- 241.60	517.97 +/- 316.52	491.47 +/- 221.00	458.74 +/- 158.60	0.0554	0.0163
ENSG00000106459	NRF1	Autophagy regulators	575	715.10 +/- 214.64	716.42 +/- 225.88	717.79 +/- 224.42	711.09 +/- 193.16	0.9494	0.8082
ENSG00000101412	E2F1	Autophagy regulators	575	54.05 +/- 22.73	55.69 +/- 24.02	54.65 +/- 23.84	51.81 +/- 20.04	0.2246	0.0948
ENSG00000160741	CRTC2	Autophagy regulators	575	748.11 +/- 250.19	753.73 +/- 276.56	755.66 +/- 255.75	734.97 +/- 215.29	0.6709	0.4633
ENSG00000150907	FOXO1	Autophagy regulators	575	1634.47 +/- 828.15	1690.44 +/- 951.86	1690.01 +/- 862.43	1523.26 +/- 630.33	0.0741	0.0479
ENSG00000116044	NFE2L2	Autophagy regulators	575	1626.59 +/- 500.58	1680.80 +/- 562.61	1645.18 +/- 514.55	1554.07 +/- 406.04	0.0378	0.0131
ENSG00000177606	JUN	Autophagy regulators	575	6711.49 +/- 2705.05	7022.68 +/- 3168.45	6812.59 +/- 2570.00	6300.81 +/- 2263.21	0.0268	0.0089
ENSG00000082641	NFE2L1	Autophagy regulators	575	42475.05 +/- 14115.05	42293.34 +/- 14899.33	42799.96 +/- 15329.47	42330.91 +/- 11948.49	0.9264	0.9797
ENSG00000118503	TNFAIP3	Autophagy regulators	575	240.31 +/- 112.33	244.02 +/- 113.91	251.00 +/- 129.61	225.92 +/- 88.74	0.0780	0.1144
ENSG00000175197	DDIT3	Autophagy regulators	575	432.37 +/- 135.99	427.11 +/- 142.81	431.66 +/- 137.32	438.30 +/- 127.95	0.7212	0.4212
ENSG00000185122	HSF1	Autophagy regulators	575	2997.12 +/- 1073.20	2956.46 +/- 1137.49	3063.64 +/- 1115.17	2971.06 +/- 961.14	0.5706	0.8955
ENSG00000089163	SIRT4	Autophagy regulators	575	146.33 +/- 49.35	144.67 +/- 51.57	145.56 +/- 49.79	148.76 +/- 46.75	0.6961	0.4182
ENSG00000171223	JUNB	Autophagy regulators	575	295.10 +/- 265.73	298.81 +/- 272.93	308.06 +/- 279.20	278.45 +/- 244.33	0.5367	0.4532
ENSG00000185591	SP1	Autophagy regulators	575	2112.39 +/- 693.68	2125.92 +/- 796.66	2114.43 +/- 700.60	2096.90 +/- 568.74	0.9187	0.6825
ENSG00000204060	FOXO6	Autophagy regulators	575	64.03 +/- 37.95	65.30 +/- 37.99	70.35 +/- 42.22	56.45 +/- 31.80	0.0013	0.0221
ENSG00000176697	BDNF	Autophagy regulators	575	64.22 +/- 107.16	77.14 +/- 123.03	70.81 +/- 122.88	44.78 +/- 61.32	0.0072	0.0030
ENSG00000100219	XBP1	Autophagy regulators	575	798.46 +/- 240.55	799.96 +/- 257.45	817.13 +/- 246.57	778.31 +/- 215.40	0.2854	0.3778
ENSG00000115415	STAT1	Autophagy regulators	575	1966.01 +/- 2114.82	2131.73 +/- 2745.69	2024.03 +/- 2193.77	1743.13 +/- 1023.76	0.1783	0.0720
ENSG00000155846	PPARGC1B	Autophagy regulators	575	683.02 +/- 287.19	694.92 +/- 310.83	682.08 +/- 277.46	672.14 +/- 272.99	0.7394	0.4382
ENSG00000162772	ATF3	Autophagy regulators	575	304.20 +/- 545.45	346.50 +/- 641.12	299.95 +/- 492.71	266.38 +/- 489.09	0.3534	0.1508
ENSG00000012504	NR1H4	Autophagy regulators	575	0.34 +/- 0.66	0.44 +/- 0.78	0.26 +/- 0.55	0.33 +/- 0.63	0.0284	0.1166
ENSG00000148516	ZEB1	Autophagy regulators	575	4342.03 +/- 1496.78	4254.21 +/- 1674.19	4407.05 +/- 1562.78	4364.39 +/- 1220.59	0.5886	0.4725
ENSG00000077463	SIRT6	Autophagy regulators	575	164.17 +/- 62.47	163.36 +/- 65.80	170.19 +/- 64.33	158.94 +/- 56.74	0.2061	0.4878
ENSG00000172216	CEBPB	Autophagy regulators	575	5197.20 +/- 2295.44	5285.16 +/- 2457.71	5408.32 +/- 2386.70	4898.57 +/- 1996.89	0.0758	0.0989
ENSG00000128272	ATF4	Autophagy regulators	575	8669.45 +/- 2354.53	8579.10 +/- 2346.15	8689.53 +/- 2522.86	8739.25 +/- 2192.39	0.7935	0.5063
ENSG00000136944	LMX1B	Autophagy regulators	575	4.55 +/- 3.70	4.48 +/- 3.28	5.04 +/- 4.72	4.13 +/- 2.79	0.0518	0.3520
ENSG00000187531	SIRT7	Autophagy regulators	575	224.23 +/- 86.87	230.51 +/- 97.86	232.68 +/- 87.30	209.54 +/- 72.14	0.0155	0.0179
ENSG00000079999	KEAP1	Autophagy regulators	575	4486.73 +/- 1526.43	4450.13 +/- 1612.52	4572.78 +/- 1568.17	4437.08 +/- 1394.66	0.6311	0.9323
ENSG00000125347	IRF1	Autophagy regulators	575	298.57 +/- 196.26	313.65 +/- 264.25	304.82 +/- 173.68	277.30 +/- 124.12	0.1673	0.0697
ENSG00000144895	EIF2A	Autophagy regulators	575	2567.84 +/- 747.76	2506.35 +/- 814.08	2576.13 +/- 764.81	2620.72 +/- 655.37	0.3211	0.1347
ENSG00000135547	HEY2	Autophagy regulators	575	342.79 +/- 120.93	345.01 +/- 132.29	339.29 +/- 121.76	344.08 +/- 108.13	0.8844	0.9409
ENSG00000115966	ATF2	Autophagy regulators	575	909.11 +/- 280.76	893.63 +/- 308.07	912.73 +/- 290.93	920.89 +/- 239.71	0.6226	0.3427
ENSG00000160877	NACC1	Autophagy regulators	575	632.46 +/- 245.93	631.08 +/- 259.15	651.80 +/- 259.92	614.48 +/- 216.07	0.3302	0.5080
ENSG00000096717	SIRT1	Autophagy regulators	575	836.07 +/- 279.86	830.36 +/- 322.23	838.60 +/- 281.94	839.23 +/- 229.23	0.9421	0.7570
ENSG00000189298	ZKSCAN3	Autophagy regulators	575	113.97 +/- 40.43	112.95 +/- 42.11	114.72 +/- 41.90	114.22 +/- 37.30	0.9067	0.7581
ENSG00000118689	FOXO3	Autophagy regulators	575	4547.29 +/- 1853.92	4479.21 +/- 2074.32	4546.97 +/- 1859.28	4615.35 +/- 1607.05	0.7731	0.4729
ENSG00000140009	ESR2	Autophagy regulators	575	22.89 +/- 11.10	23.30 +/- 11.51	23.55 +/- 12.23	21.83 +/- 9.35	0.2623	0.1964
ENSG00000118217	ATF6	Autophagy regulators	575	712.97 +/- 210.71	707.66 +/- 230.49	719.27 +/- 223.05	711.95 +/- 175.37	0.8624	0.8429
ENSG00000162761	LMX1A	Autophagy regulators	575	14.94 +/- 7.42	14.84 +/- 7.55	15.15 +/- 7.74	14.84 +/- 6.98	0.8978	0.9942
ENSG00000132005	RFX1	Autophagy regulators	575	282.70 +/- 124.46	274.56 +/- 127.30	288.48 +/- 129.22	285.02 +/- 116.75	0.5233	0.4120
ENSG00000184481	FOXO4	Autophagy regulators	575	3400.03 +/- 1196.07	3312.42 +/- 1241.10	3406.91 +/- 1265.46	3480.29 +/- 1073.03	0.3883	0.1699
ENSG00000068305	MEF2A	Autophagy regulators	575	4916.37 +/- 1638.54	4837.15 +/- 1823.19	4954.37 +/- 1712.74	4957.18 +/- 1348.71	0.7165	0.4744
ENSG00000102882	MAPK3	Autophagy regulators	575	790.71 +/- 272.48	793.17 +/- 281.50	799.31 +/- 286.91	779.66 +/- 248.55	0.7707	0.6274
ENSG00000118260	CREB1	Autophagy regulators	575	620.82 +/- 206.15	616.22 +/- 228.81	622.39 +/- 213.44	623.82 +/- 173.33	0.9294	0.7187
ENSG00000177169	ULK1	Autophagy regulators, Mitophagy	575	2473.06 +/- 1020.77	2472.82 +/- 1119.41	2553.61 +/- 1067.71	2392.73 +/- 855.48	0.3040	0.4419
ENSG00000161011	SQSTM1	Autophagy regulators, Mitophagy	575	15033.98 +/- 4679.37	15216.53 +/- 4980.86	15298.81 +/- 4764.83	14587.55 +/- 4256.44	0.2658	0.1882
ENSG00000124523	SIRT5	Autophagy regulators, Mitophagy	575	1477.89 +/- 496.09	1426.32 +/- 498.22	1467.28 +/- 512.59	1539.81 +/- 472.53	0.0762	0.0250
ENSG00000142082	SIRT3	Autophagy regulators, Mitophagy	575	1001.39 +/- 333.02	983.94 +/- 343.77	1008.32 +/- 342.67	1011.81 +/- 312.82	0.6726	0.4136
ENSG00000068903	SIRT2	Autophagy regulators, Mitophagy	575	7300.37 +/- 2387.54	7349.13 +/- 2623.30	7306.60 +/- 2400.32	7245.64 +/- 2125.63	0.9134	0.6718
ENSG00000100030	MAPK1	Autophagy regulators, Mitophagy	575	2744.01 +/- 878.19	2750.77 +/- 953.12	2806.29 +/- 935.31	2674.99 +/- 728.33	0.3397	0.3979
ENSG00000181449	SOX2	Autophagy regulators, Mitophagy	575	1.80 +/- 2.18	1.93 +/- 2.88	1.74 +/- 1.84	1.72 +/- 1.60	0.5965	0.3508
ENSG00000105967	TFEC	Autophagy regulators, Mitophagy	575	11.94 +/- 13.50	13.77 +/- 19.63	11.65 +/- 11.05	10.40 +/- 6.09	0.0474	0.0145
ENSG00000168610	STAT3	Autophagy regulators, mTOR and upstream pathways	575	4907.30 +/- 1764.06	5118.34 +/- 1958.54	4962.85 +/- 1828.84	4641.82 +/- 1439.83	0.0261	0.0081
ENSG00000100811	YY1	Autophagy regulators, mTOR and upstream pathways	575	2233.25 +/- 623.57	2202.23 +/- 672.09	2272.47 +/- 672.29	2224.88 +/- 514.46	0.5315	0.7239
ENSG00000100644	HIF1A	Autophagy regulators, mTOR and upstream pathways	575	483.24 +/- 160.86	474.59 +/- 175.55	488.40 +/- 166.66	486.69 +/- 138.64	0.6583	0.4627
ENSG00000068323	TFE3	Autophagy regulators, Mitophagy, mTOR and upstream pathw	575	733.48 +/- 231.03	752.12 +/- 244.29	750.00 +/- 246.83	698.42 +/- 195.59	0.0358	0.0227
ENSG00000112561	TFEB	Autophagy regulators, Mitophagy, mTOR and upstream pathw	575	1930.71 +/- 799.81	1947.29 +/- 888.45	1980.91 +/- 816.44	1864.01 +/- 680.71	0.3379	0.3079
ENSG00000187098	MITF	Autophagy regulators, Mitophagy, mTOR and upstream pathw	575	2653.58 +/- 893.68	2602.46 +/- 959.79	2644.84 +/- 941.41	2713.18 +/- 768.95	0.4738	0.2256
ENSG00000198959	TGM2	Mitophagy	575	860.14 +/- 375.45	848.88 +/- 396.14	879.32 +/- 406.49	852.18 +/- 318.93	0.6849	0.9326
ENSG00000158828	PINK1	Mitophagy	575	11943.63 +/- 4761.71	11791.15 +/- 5008.96	12382.04 +/- 4950.18	11656.90 +/- 4287.03	0.2842	0.7811
ENSG00000094631	HDAC6	Mitophagy	575	1580.47 +/- 531.93	1616.68 +/- 593.67	1597.14 +/- 544.49	1527.79 +/- 445.95	0.2284	0.1019
ENSG00000104765	BNIP3L	Mitophagy	575	749.00 +/- 232.90	758.13 +/- 250.01	763.59 +/- 255.18	725.33 +/- 186.69	0.2201	0.1679
ENSG00000112062	MAPK14	Mitophagy	575	1869.52 +/- 552.70	1883.77 +/- 612.12	1874.93 +/- 563.56	1849.92 +/- 476.53	0.8246	0.5492
ENSG00000123240	OPTN	Mitophagy	575	22010.93 +/- 6305.80	21041.59 +/- 6501.93	21951.09 +/- 6531.37	23035.07 +/- 5726.05	0.0081	0.0019
ENSG00000136436	CALCOCO2	Mitophagy	575	4293.13 +/- 1199.38	4307.02 +/- 1336.23	4275.76 +/- 1258.99	4296.69 +/- 980.49	0.9669	0.9332
ENSG00000184584	STING1	Mitophagy	575	308.29 +/- 128.00	303.61 +/- 138.39	315.05 +/- 133.28	306.17 +/- 111.14	0.6565	0.8458
ENSG00000106052	TAX1BP1	Mitophagy	575	3164.14 +/- 944.83	3137.68 +/- 1047.05	3182.32 +/- 995.24	3172.30 +/- 775.00	0.8894	0.7206
ENSG00000183735	TBK1	Mitophagy	575	623.84 +/- 202.54	610.24 +/- 222.34	631.24 +/- 211.25	629.97 +/- 170.88	0.5245	0.3413
ENSG00000111540	RAB5B	Mitophagy	575	5012.75 +/- 1461.20	5121.60 +/- 1629.05	4972.69 +/- 1493.99	4944.54 +/- 1235.48	0.4450	0.2363
ENSG00000144566	RAB5A	Mitophagy	575	1046.26 +/- 305.75	1026.73 +/- 338.23	1058.07 +/- 323.08	1053.88 +/- 249.36	0.5537	0.3859
ENSG00000171552	BCL2L1	Mitophagy	575	663.02 +/- 219.66	664.71 +/- 235.20	672.44 +/- 235.31	651.93 +/- 185.43	0.6534	0.5690
ENSG00000198742	SMURF1	Mitophagy	575	559.84 +/- 228.67	556.97 +/- 261.57	574.63 +/- 226.74	547.91 +/- 193.15	0.5085	0.6971
ENSG00000126581	BECN1	Mitophagy	575	2236.49 +/- 653.72	2162.21 +/- 668.88	2263.01 +/- 689.52	2283.85 +/- 596.07	0.1504	0.0688
ENSG00000023287	RB1CC1	Mitophagy	575	2894.84 +/- 1013.30	2780.91 +/- 1111.52	2897.70 +/- 1061.43	3005.33 +/- 839.51	0.0953	0.0301
ENSG00000138592	USP8	Mitophagy	575	1106.54 +/- 329.29	1081.28 +/- 357.40	1110.55 +/- 349.45	1127.65 +/- 274.91	0.3795	0.1685
ENSG00000075785	RAB7A	Mitophagy	575	7091.28 +/- 1900.42	7006.41 +/- 2040.71	7231.19 +/- 1998.40	7035.79 +/- 1639.34	0.4534	0.8814
ENSG00000108774	RAB5C	Mitophagy	575	225.24 +/- 78.76	228.63 +/- 84.32	225.35 +/- 78.35	221.77 +/- 73.54	0.6954	0.3940
ENSG00000146729	NIPSNAP2	Mitophagy	575	13973.28 +/- 3950.98	13333.27 +/- 4110.35	14115.14 +/- 4194.96	14468.09 +/- 3439.80	0.0157	0.0049
ENSG00000087470	DNM1L	Mitophagy	575	2672.31 +/- 876.89	2522.53 +/- 915.82	2664.48 +/- 881.09	2829.12 +/- 808.06	0.0027	0.0006
ENSG00000163071	SPATA18	Mitophagy	575	95.71 +/- 56.40	103.58 +/- 61.87	101.99 +/- 58.39	81.59 +/- 45.17	0.0001	0.0001
ENSG00000104946	TBC1D17	Mitophagy	575	2916.09 +/- 1102.62	3012.28 +/- 1221.72	2943.79 +/- 1091.07	2792.70 +/- 976.27	0.1368	0.0512
ENSG00000090432	MUL1	Mitophagy	575	1022.17 +/- 320.53	1009.21 +/- 333.88	1028.35 +/- 329.47	1028.89 +/- 298.43	0.7921	0.5490
ENSG00000143384	MCL1	Mitophagy	575	8611.87 +/- 2646.48	8792.46 +/- 2992.60	8569.21 +/- 2645.50	8474.89 +/- 2255.56	0.4842	0.2408
ENSG00000116688	MFN2	Mitophagy	575	14394.23 +/- 5029.47	13974.69 +/- 5247.28	14376.92 +/- 5205.35	14828.91 +/- 4600.80	0.2513	0.0965
ENSG00000198836	OPA1	Mitophagy	575	3310.89 +/- 1114.00	3156.15 +/- 1191.01	3303.28 +/- 1135.72	3472.43 +/- 989.10	0.0207	0.0054
ENSG00000188906	LRRK2	Mitophagy	575	747.66 +/- 335.67	763.95 +/- 402.43	756.04 +/- 347.09	723.08 +/- 236.87	0.4503	0.2336

ENSG00000168958	MFF	Mitophagy	575	1790.36 +/- 488.24	1729.50 +/- 504.30	1801.37 +/- 515.92	1839.90 +/- 436.97	0.0802	0.0268
ENSG00000100335	MIEF1	Mitophagy	575	675.92 +/- 204.38	657.39 +/- 215.06	684.90 +/- 217.33	685.38 +/- 178.26	0.3091	0.1809
ENSG00000215475	SIAH3	Mitophagy	575	2.48 +/- 2.98	2.73 +/- 3.46	2.37 +/- 2.98	2.35 +/- 2.40	0.3828	0.2206
ENSG00000167085	PHB1	Mitophagy	575	3143.08 +/- 922.36	3113.60 +/- 980.68	3143.73 +/- 928.84	3171.76 +/- 857.64	0.8271	0.5377
ENSG00000138095	LRPPRC	Mitophagy	575	8651.05 +/- 2815.53	8382.35 +/- 3033.26	8623.65 +/- 2906.98	8945.76 +/- 2461.67	0.1451	0.0501
ENSG00000069509	FUNDC1	Mitophagy	575	274.64 +/- 75.72	274.24 +/- 80.41	272.51 +/- 75.55	277.17 +/- 71.26	0.8309	0.7052
ENSG00000165280	VCP	Mitophagy	575	9318.67 +/- 2842.46	9497.59 +/- 3058.51	9337.52 +/- 2903.43	9121.82 +/- 2544.46	0.4311	0.1960
ENSG00000215021	PHB2	Mitophagy	575	4817.20 +/- 1365.65	4644.19 +/- 1354.05	4844.92 +/- 1448.71	4961.59 +/- 1276.92	0.0708	0.0229
ENSG00000223802	CERS1	Mitophagy	575	94.10 +/- 42.08	95.59 +/- 45.98	93.20 +/- 43.26	93.52 +/- 36.65	0.8335	0.6301
ENSG00000135093	USP30	Mitophagy	575	601.82 +/- 176.92	598.93 +/- 189.46	609.28 +/- 188.07	597.23 +/- 151.31	0.7711	0.9244
ENSG00000204390	HSPA1L	Mitophagy	575	215.44 +/- 80.95	222.00 +/- 88.40	215.47 +/- 79.58	208.89 +/- 74.16	0.2851	0.1130
ENSG00000121749	TBC1D15	Mitophagy	575	1040.79 +/- 314.78	1053.14 +/- 352.89	1044.58 +/- 331.54	1024.71 +/- 252.09	0.6636	0.3772
ENSG00000072310	SREBF1	Mitophagy	575	1219.82 +/- 687.82	1233.43 +/- 675.44	1283.54 +/- 735.11	1142.56 +/- 645.98	0.1258	0.1955
ENSG00000185651	UBE2L3	Mitophagy	575	1902.92 +/- 513.32	1913.14 +/- 542.14	1941.72 +/- 544.95	1853.96 +/- 445.55	0.2327	0.2588
ENSG00000214253	FIS1	Mitophagy	575	3744.99 +/- 1161.25	3662.46 +/- 1169.92	3767.63 +/- 1207.35	3804.45 +/- 1105.65	0.4635	0.2320
ENSG00000177427	MIEF2	Mitophagy	575	1383.81 +/- 492.63	1359.79 +/- 497.54	1407.86 +/- 518.16	1383.67 +/- 461.99	0.6346	0.6365
ENSG00000181220	ZNF746	Mitophagy	575	278.52 +/- 92.25	273.34 +/- 97.55	285.36 +/- 98.40	276.84 +/- 79.67	0.4232	0.7123
ENSG00000099968	BCL2L13	Mitophagy	575	3404.50 +/- 1092.03	3312.23 +/- 1139.99	3461.96 +/- 1194.83	3438.82 +/- 922.20	0.3532	0.2576
ENSG00000067900	ROCK1	Mitophagy	575	858.51 +/- 267.11	841.64 +/- 286.89	867.74 +/- 280.36	866.06 +/- 231.33	0.5651	0.3720
ENSG00000177889	UBE2N	Mitophagy	575	1972.85 +/- 528.17	1980.15 +/- 585.19	1992.34 +/- 534.44	1946.09 +/- 459.25	0.6742	0.5279
ENSG00000135655	USP15	Mitophagy	575	3455.36 +/- 1083.40	3378.51 +/- 1184.65	3455.41 +/- 1105.96	3531.77 +/- 946.83	0.3843	0.1665
ENSG00000184117	NIPSNAP1	Mitophagy	575	65.93 +/- 35.20	64.66 +/- 34.35	66.89 +/- 36.46	66.23 +/- 34.90	0.8178	0.6646
ENSG00000198911	SREBF2	Mitophagy	575	1543.38 +/- 534.05	1547.30 +/- 586.98	1573.18 +/- 557.80	1509.70 +/- 448.55	0.5045	0.4904
ENSG00000116288	PARK7	Mitophagy	575	6022.32 +/- 1571.72	6120.06 +/- 1709.72	6031.20 +/- 1588.96	5916.20 +/- 1403.67	0.4456	0.2046
ENSG00000176171	BNIP3	Mitophagy	575	4232.10 +/- 1411.62	4252.63 +/- 1531.18	4319.15 +/- 1467.55	4124.64 +/- 1217.75	0.3907	0.3746
ENSG00000100225	FBXO7	Mitophagy	575	1006.37 +/- 298.04	1011.01 +/- 324.89	1011.06 +/- 309.37	997.06 +/- 257.10	0.8692	0.6470
ENSG00000156735	BAG4	Mitophagy	575	324.20 +/- 96.21	326.30 +/- 107.01	322.53 +/- 99.80	323.79 +/- 80.36	0.9268	0.7994
ENSG00000109332	UBE2D3	Mitophagy	575	6989.01 +/- 1936.35	6991.84 +/- 2135.08	7015.69 +/- 2021.81	6959.53 +/- 1626.03	0.9603	0.8702
ENSG00000118369	USP35	Mitophagy	575	173.24 +/- 67.83	166.32 +/- 68.42	179.25 +/- 72.64	174.11 +/- 61.72	0.1718	0.2625
ENSG00000185345	PRKN	Mitophagy	575	1143.49 +/- 380.44	1111.31 +/- 396.45	1136.04 +/- 396.04	1182.93 +/- 345.11	0.1736	0.0653
ENSG00000138942	RNF185	Mitophagy	575	946.29 +/- 276.57	949.28 +/- 301.26	953.24 +/- 286.08	936.38 +/- 240.00	0.8232	0.6480
ENSG00000131508	UBE2D2	Mitophagy	575	2920.22 +/- 799.55	2873.77 +/- 837.24	2941.73 +/- 854.83	2944.90 +/- 699.93	0.6175	0.3849
ENSG00000196683	TOMM7	Mitophagy	575	4483.30 +/- 1298.33	4635.60 +/- 1369.50	4348.43 +/- 1359.02	4466.65 +/- 1145.17	0.0937	0.2041
ENSG00000171109	MFN1	Mitophagy	575	1396.18 +/- 415.76	1362.75 +/- 454.14	1396.07 +/- 429.03	1429.55 +/- 357.79	0.2911	0.1160
ENSG00000115317	HTRA2	Mitophagy	575	467.26 +/- 150.30	468.83 +/- 156.36	474.60 +/- 158.35	458.36 +/- 135.47	0.5630	0.4952
ENSG00000159363	ATP13A2	Mitophagy	575	440.22 +/- 170.04	446.95 +/- 182.93	452.07 +/- 178.63	421.68 +/- 145.50	0.1727	0.1456
ENSG00000110497	AMBRA1	Mitophagy, mTOR and upstream pathways	575	495.55 +/- 157.83	497.75 +/- 169.78	505.75 +/- 170.45	483.16 +/- 129.90	0.3643	0.3652
ENSG00000130766	SESN2	Mitophagy, mTOR and upstream pathways	575	160.07 +/- 81.20	161.15 +/- 85.67	168.64 +/- 88.62	150.43 +/- 67.00	0.0870	0.1955
ENSG00000154803	FLCN	Mitophagy, mTOR and upstream pathways	575	1470.43 +/- 541.60	1528.85 +/- 621.02	1458.23 +/- 530.00	1424.50 +/- 459.69	0.1573	0.0594
ENSG00000106615	RHEB	Mitophagy, mTOR and upstream pathways	575	1934.25 +/- 542.15	1983.51 +/- 597.46	1951.17 +/- 556.40	1868.31 +/- 459.94	0.0999	0.0374
ENSG00000217128	FNIP1	Mitophagy, mTOR and upstream pathways	575	1459.75 +/- 485.99	1467.88 +/- 544.89	1440.84 +/- 488.18	1470.56 +/- 419.02	0.8034	0.9564
ENSG00000106617	PRKAG2	mTOR and upstream pathways	575	266.13 +/- 130.18	242.53 +/- 102.81	267.54 +/- 110.19	288.19 +/- 165.00	0.0026	0.0006
ENSG00000108840	HDAC5	mTOR and upstream pathways	575	3878.28 +/- 1394.40	4007.14 +/- 1530.20	3909.27 +/- 1400.65	3719.11 +/- 1228.46	0.1207	0.0431
ENSG00000115592	PRKAG3	mTOR and upstream pathways	575	2243.52 +/- 1251.71	2399.25 +/- 1338.61	2351.65 +/- 1293.57	1980.47 +/- 1071.05	0.0015	0.0010
ENSG00000103319	EEF2K	mTOR and upstream pathways	575	3403.86 +/- 1180.93	3492.40 +/- 1295.96	3497.26 +/- 1228.76	3222.38 +/- 980.19	0.0329	0.0250
ENSG00000276231	PIK3R6	mTOR and upstream pathways	575	16.60 +/- 10.74	15.81 +/- 11.43	17.18 +/- 10.80	16.82 +/- 9.95	0.4332	0.3580
ENSG00000119487	MAPKAP1	mTOR and upstream pathways	575	2424.84 +/- 744.85	2319.24 +/- 735.98	2458.49 +/- 786.10	2496.23 +/- 702.22	0.0497	0.0200
ENSG00000082146	STRADB	mTOR and upstream pathways	575	2609.40 +/- 798.79	2475.05 +/- 813.00	2660.65 +/- 859.58	2691.80 +/- 702.58	0.0160	0.0079
ENSG00000181929	PRKAG1	mTOR and upstream pathways	575	2728.36 +/- 806.13	2586.40 +/- 803.04	2717.80 +/- 823.37	2880.14 +/- 768.16	0.0016	0.0003
ENSG00000102547	CAB39L	mTOR and upstream pathways	575	339.68 +/- 205.03	379.31 +/- 255.12	343.29 +/- 196.96	296.65 +/- 139.08	0.0004	<.0001
ENSG00000158186	MRAS	mTOR and upstream pathways	575	1561.36 +/- 636.48	1686.44 +/- 679.62	1609.90 +/- 700.11	1388.38 +/- 468.76	<.0001	<.0001
ENSG00000149212	SESN3	mTOR and upstream pathways	575	630.26 +/- 345.05	681.47 +/- 363.98	654.74 +/- 397.44	554.83 +/- 243.14	0.0007	0.0003
ENSG00000142208	AKT1	mTOR and upstream pathways	575	1850.49 +/- 628.63	1792.68 +/- 638.27	1898.63 +/- 670.02	1859.88 +/- 572.40	0.2489	0.2968
ENSG00000103091	WDR59	mTOR and upstream pathways	575	1181.02 +/- 345.17	1204.39 +/- 378.20	1187.10 +/- 365.22	1151.70 +/- 284.47	0.3140	0.1353
ENSG00000127580	WDR24	mTOR and upstream pathways	575	358.29 +/- 142.20	365.88 +/- 155.00	368.82 +/- 142.67	340.21 +/- 126.54	0.0953	0.0770
ENSG00000118046	STK11	mTOR and upstream pathways	575	3220.22 +/- 1214.81	3269.28 +/- 1341.80	3301.18 +/- 1202.74	3090.47 +/- 1082.37	0.1871	0.1495
ENSG00000117461	PIK3R3	mTOR and upstream pathways	575	284.41 +/- 114.07	284.42 +/- 116.26	286.05 +/- 122.01	282.77 +/- 103.77	0.9613	0.8871
ENSG00000134248	LAMTOR5	mTOR and upstream pathways	575	2343.89 +/- 594.43	2339.86 +/- 631.08	2330.91 +/- 605.75	2360.86 +/- 546.18	0.8798	0.7295
ENSG00000106070	GRB10	mTOR and upstream pathways	575	3537.92 +/- 1599.93	3309.65 +/- 1563.37	3582.82 +/- 1657.56	3720.09 +/- 1557.70	0.0380	0.0120
ENSG00000114388	NPRL2	mTOR and upstream pathways	575	731.03 +/- 237.71	744.92 +/- 256.25	740.63 +/- 238.64	707.63 +/- 216.11	0.2437	0.1246
ENSG00000174775	HRAS	mTOR and upstream pathways	575	1071.26 +/- 405.11	1077.72 +/- 441.07	1094.51 +/- 405.32	1041.59 +/- 365.95	0.4258	0.3824
ENSG00000164603	BMT2	mTOR and upstream pathways	575	206.53 +/- 67.22	203.94 +/- 73.39	207.66 +/- 69.54	207.97 +/- 58.08	0.8087	0.5580
ENSG00000131791	PRKAB2	mTOR and upstream pathways	575	6526.77 +/- 2262.98	6254.68 +/- 2497.19	6523.34 +/- 2316.73	6800.86 +/- 1915.71	0.0612	0.0181
ENSG00000111203	ITFG2	mTOR and upstream pathways	575	631.97 +/- 187.49	652.31 +/- 211.01	628.46 +/- 190.61	615.23 +/- 155.91	0.1462	0.0529
ENSG00000155506	LARP1	mTOR and upstream pathways	575	3953.97 +/- 1303.96	3956.61 +/- 1388.89	4010.23 +/- 1416.29	3895.07 +/- 1085.53	0.6880	0.6438
ENSG00000141564	RPTOR	mTOR and upstream pathways	575	1153.93 +/- 438.75	1157.85 +/- 471.96	1178.80 +/- 454.28	1125.16 +/- 386.12	0.4833	0.4656
ENSG00000165699	TSC1	mTOR and upstream pathways	575	2238.64 +/- 710.95	2162.31 +/- 759.68	2236.92 +/- 741.55	2316.30 +/- 618.97	0.1056	0.0339
ENSG00000187840	EIF4EBP1	mTOR and upstream pathways	575	412.38 +/- 208.52	371.38 +/- 169.74	418.49 +/- 212.86	447.06 +/- 231.90	0.0015	0.0004
ENSG00000103148	NPRL3	mTOR and upstream pathways	575	837.48 +/- 323.13	871.55 +/- 362.39	844.60 +/- 322.36	796.48 +/- 276.28	0.0702	0.0228
ENSG00000103197	TSC2	mTOR and upstream pathways	575	2434.85 +/- 943.43	2469.52 +/- 1036.00	2476.65 +/- 996.37	2358.55 +/- 778.20	0.3892	0.2496
ENSG00000108443	RPS6KB1	mTOR and upstream pathways	575	1551.15 +/- 519.57	1526.58 +/- 582.00	1582.92 +/- 539.15	1543.84 +/- 426.58	0.5543	0.7465
ENSG00000105851	PIK3CG	mTOR and upstream pathways	575	10.33 +/- 6.41	9.92 +/- 6.86	10.45 +/- 6.51	10.63 +/- 5.83	0.5371	0.2836
ENSG00000163507	CIP2A	mTOR and upstream pathways	575	38.84 +/- 17.35	35.98 +/- 16.20	39.17 +/- 17.84	41.35 +/- 17.64	0.0095	0.0024
ENSG00000159217	IGF2BP1	mTOR and upstream pathways	575	0.53 +/- 1.08	0.64 +/- 1.40	0.56 +/- 1.04	0.38 +/- 0.66	0.0492	0.0165
ENSG00000085415	SEH1L	mTOR and upstream pathways	575	656.02 +/- 187.29	666.61 +/- 207.63	657.49 +/- 192.08	644.01 +/- 159.30	0.4945	0.2380
ENSG00000157020	SEC13	mTOR and upstream pathways	575	1637.95 +/- 485.25	1628.16 +/- 501.82	1665.84 +/- 509.92	1619.81 +/- 442.59	0.6132	0.8653
ENSG00000213281	NRAS	mTOR and upstream pathways	575	527.11 +/- 176.66	518.27 +/- 188.60	532.89 +/- 185.78	530.14 +/- 154.17	0.6915	0.5119
ENSG00000100726	TELO2	mTOR and upstream pathways	575	706.26 +/- 283.80	716.68 +/- 310.41	721.15 +/- 286.03	681.01 +/- 251.82	0.3162	0.2185
ENSG00000025039	RRAGD	mTOR and upstream pathways	575	5558.05 +/- 1680.91	5544.38 +/- 1846.87	5599.33 +/- 1764.65	5530.37 +/- 1407.64	0.9140	0.9346
ENSG00000135932	CAB39	mTOR and upstream pathways	575	9420.33 +/- 3076.86	9466.85 +/- 3395.50	9583.41 +/- 3282.51	9210.98 +/- 2476.66	0.4798	0.4155
ENSG00000134324	LPIN1	mTOR and upstream pathways	575	5598.27 +/- 1913.75	5398.84 +/- 1887.19	5710.97 +/- 1915.81	5683.96 +/- 1932.19	0.2099	0.1454
ENSG00000052795	FNIP2	mTOR and upstream pathways	575	728.64 +/- 342.74	710.79 +/- 359.22	756.57 +/- 413.68	718.48 +/- 228.73	0.3755	0.8280
ENSG00000166167	BTRC	mTOR and upstream pathways	575	1034.59 +/- 304.40	1030.55 +/- 333.37	1029.09 +/- 317.83	1044.11 +/- 258.37	0.8681	0.6631
ENSG00000162409	PRKAA2	mTOR and upstream pathways	575	8923.59 +/- 3048.08	8759.99 +/- 3354.23	8964.69 +/- 3185.52	9045.23 +/- 2556.98	0.6413	0.3605
ENSG00000198793	MTOR	mTOR and upstream pathways	575	1956.81 +/- 702.69	1900.83 +/- 745.57	1999.02 +/- 748.21	1970.28 +/- 605.01	0.3731	0.3347
ENSG00000186654	PRR5	mTOR and upstream pathways	575	16.49 +/- 12.68	17.74 +/- 13.06	17.17 +/- 15.34	14.56 +/- 8.53	0.0320	0.0138
ENSG00000205464	ATP6AP1L	mTOR and upstream pathways	575	251.03 +/- 90.84	250.30 +/- 90.01	251.20 +/- 93.94	251.59 +/- 88.97	0.9899	0.8897
ENSG00000109270	LAMTOR3	mTOR and upstream pathways	575	1009.71 +/- 300.91	1012.66 +/- 330.02	1013.86 +/- 315.82	1002.62 +/- 252.89	0.9227	0.7441
ENSG00000116977	LGALS8	mTOR and upstream pathways	575	432.28 +/- 142.11	419.33 +/- 147.88	441.14 +/- 154.70	436.30 +/- 121.43	0.2890	0.2437
ENSG00000133056	PIK3C2B	mTOR and upstream pathways	575	1419.04 +/- 529.68	1363.58 +/- 566.70	1422.61 +/- 559.05	1470.64 +/- 453.18	0.1405	0.0479
ENSG00000239282	CASTOR1	mTOR and upstream pathways	575	2.29 +/- 1.89	2.07 +/- 1.92	2.31 +/- 1.88	2.48 +/- 1.86	0.1084	0.0357
ENSG00000101407	TTI1	mTOR and upstream pathways	575	538.84 +/- 166.86	542.94 +/- 182.29	544.07 +/- 175.33	529.54 +/- 140.52	0.6380	0.4318
ENSG00000080546	SESN1	mTOR and upstream pathways	575	8798.32 +/- 2959.06	8587.39 +/- 3292.64	8780.10 +/- 2945.33	9026.37 +/- 2599.44	0.3474	0.1467
ENSG00000111725	PRKAB1	mTOR and upstream pathways	575	412.65 +/- 129.00	401.40 +/- 134.45	421.84 +/- 138.83	414.65 +/- 111.96	0.2909	0.3164
ENSG00000133703	KRAS	mTOR and upstream pathways	575	496.69 +/- 167.28	489.79 +/- 187.44	501.52 +/- 169.79	498.72 +/- 142.27	0.7740	0.6020
ENSG00000132356	PRKAA1	mTOR and upstream pathways	575	1620.27 +/- 561.28	1549.71 +/- 614.69	1611.77 +/- 557.41	1698.97 +/- 498.92	0.0325	0.0091
ENSG00000145675	PIK3R1	mTOR and upstream pathways	575	3456.66 +/- 1685.11	3493.01 +/- 1999.93	3484.30 +/- 1558.87	3392.85 +/- 1455.54	0.8128	0.5610
ENSG00000110931	CAMKK2	mTOR and upstream pathways	575	622.98 +/- 232.81	609.94 +/- 231.43	630.88 +/- 250.35	628.07 +/- 216.05	0.6348	0.4472
ENSG00000164654	MIOS	mTOR and upstream pathways	575	1773.04 +/- 569.56	1719.46 +/- 591.33	1788.69 +/- 598.73	1810.69 +/- 513.83	0.2630	0.1173
ENSG00000141506	PIK3R5	mTOR and upstream pathways	575	32.89 +/- 27.66	35.30 +/- 35.11	33.85 +/- 28.69	29.52 +/- 15.22	0.1029	0.0404
ENSG00000051382	PIK3CB	mTOR and upstream pathways	575	1107.02 +/- 402.91	1067.14 +/- 431.68	1127.51 +/- 433.92	1126.22 +/- 334.38	0.2465	0.1519
ENSG00000155876	RRAGA	mTOR and upstream pathways	575	2313.63 +/- 670.90	2336.48 +/- 730.03	2350.22 +/- 686.28	2254.32 +/- 588.35	0.3183	0.2306
ENSG00000091831	ESR1	mTOR and upstream pathways	575	476.01 +/- 188.02	461.99 +/- 197.00	492.57 +/- 197.12	473.39 +/- 168.21	0.2746	0.5553
ENSG00000126458	RRAS	mTOR and upstream pathways	575	446.29 +/- 189.25	459.93 +/- 196.27	471.58 +/- 196.01	407.43 +/- 168.96	0.0018	0.0065
ENSG00000175634	RPS6KB2	mTOR and upstream pathways	575	1190.11 +/- 406.37	1166.07 +/- 417.86	1221.37 +/- 427.40	1182.77 +/- 371.89	0.3938	0.6894

ENSG00000204673	AKT1S1	mTOR and upstream pathways	575	2334.73 +/- 842.37	2301.98 +/- 873.89	2401.83 +/- 879.07	2300.21 +/- 770.22	0.4013	0.9820
ENSG00000071553	ATP6AP1	mTOR and upstream pathways	575	1317.99 +/- 418.11	1327.88 +/- 450.11	1335.74 +/- 428.07	1290.40 +/- 373.35	0.5257	0.3802
ENSG00000116586	LAMTOR2	mTOR and upstream pathways	575	676.29 +/- 217.35	680.76 +/- 224.99	682.06 +/- 227.36	666.08 +/- 199.40	0.7271	0.5088
ENSG00000113575	PPP2CA	mTOR and upstream pathways	575	2790.84 +/- 968.96	2920.41 +/- 1136.62	2784.64 +/- 993.68	2668.15 +/- 719.09	0.0385	0.0107
ENSG00000068024	HDAC4	mTOR and upstream pathways	575	2395.42 +/- 926.54	2356.23 +/- 960.25	2438.39 +/- 951.38	2391.45 +/- 868.41	0.6852	0.7111
ENSG00000118162	KPTN	mTOR and upstream pathways	575	196.56 +/- 72.11	208.67 +/- 82.45	198.28 +/- 70.86	182.80 +/- 59.18	0.0019	0.0004
ENSG00000188186	LAMTOR4	mTOR and upstream pathways	575	708.79 +/- 250.51	717.77 +/- 261.66	710.49 +/- 255.83	698.15 +/- 234.17	0.7410	0.4436
ENSG00000129696	TTI2	mTOR and upstream pathways	575	179.23 +/- 52.38	178.53 +/- 54.66	182.42 +/- 54.08	176.73 +/- 48.27	0.5549	0.7366
ENSG00000121879	PIK3CA	mTOR and upstream pathways	575	1173.05 +/- 408.68	1135.68 +/- 446.01	1184.59 +/- 437.97	1198.70 +/- 331.72	0.2860	0.1316
ENSG00000154229	PRKCA	mTOR and upstream pathways	575	666.08 +/- 314.25	633.10 +/- 319.47	700.53 +/- 333.06	664.43 +/- 286.40	0.1098	0.3311
ENSG00000167965	MLST8	mTOR and upstream pathways	575	956.56 +/- 367.11	932.37 +/- 378.87	981.72 +/- 383.76	955.47 +/- 337.23	0.4211	0.5396
ENSG00000266173	STRADA	mTOR and upstream pathways	575	88.15 +/- 30.39	87.40 +/- 33.97	88.79 +/- 30.67	88.25 +/- 26.21	0.9044	0.7858
ENSG00000174206	KICS2	mTOR and upstream pathways	575	152.74 +/- 48.98	150.48 +/- 52.10	154.41 +/- 50.51	153.33 +/- 44.16	0.7209	0.5699
ENSG00000105647	PIK3R2	mTOR and upstream pathways	575	1.92 +/- 1.52	2.03 +/- 1.59	1.82 +/- 1.55	1.91 +/- 1.43	0.3873	0.4231
ENSG00000011405	PIK3C2A	mTOR and upstream pathways	575	657.61 +/- 226.20	638.88 +/- 250.91	660.59 +/- 229.92	673.25 +/- 194.11	0.3235	0.1373
ENSG00000198198	SZT2	mTOR and upstream pathways	575	1220.24 +/- 452.50	1207.55 +/- 491.78	1243.77 +/- 476.44	1209.34 +/- 383.32	0.6775	0.9703
ENSG00000171608	PIK3CD	mTOR and upstream pathways	575	76.68 +/- 45.98	78.98 +/- 56.28	79.65 +/- 47.55	71.41 +/- 29.98	0.1491	0.1065
ENSG00000112977	DAP	mTOR and upstream pathways	575	185.77 +/- 79.28	184.93 +/- 79.64	194.77 +/- 90.23	177.62 +/- 65.50	0.1041	0.3660
ENSG00000100150	DEPDC5	mTOR and upstream pathways	575	233.74 +/- 76.25	233.20 +/- 83.23	233.89 +/- 78.72	234.11 +/- 66.21	0.9927	0.9072
ENSG00000116954	RRAGC	mTOR and upstream pathways	575	226.25 +/- 66.82	229.70 +/- 71.91	227.27 +/- 69.32	221.81 +/- 58.62	0.4976	0.2485
ENSG00000135362	PRR5L	mTOR and upstream pathways	575	26.21 +/- 18.38	27.64 +/- 18.69	26.36 +/- 21.50	24.64 +/- 14.16	0.2761	0.1098
ENSG00000139144	PIK3C2G	mTOR and upstream pathways	575	0.14 +/- 0.47	0.16 +/- 0.63	0.13 +/- 0.37	0.12 +/- 0.37	0.6572	0.3793
ENSG00000155792	DEPTOR	mTOR and upstream pathways	575	4472.34 +/- 1657.99	4332.33 +/- 1757.72	4632.20 +/- 1774.86	4451.77 +/- 1411.36	0.2045	0.4829
ENSG00000117676	RPS6KA1	mTOR and upstream pathways	575	44.68 +/- 27.65	46.91 +/- 32.58	44.59 +/- 28.76	42.56 +/- 20.10	0.3059	0.1239
ENSG00000083750	RRAGB	mTOR and upstream pathways	575	486.39 +/- 145.10	485.48 +/- 158.54	483.75 +/- 150.19	489.95 +/- 125.25	0.9112	0.7629
ENSG00000118515	SGK1	mTOR and upstream pathways	575	1740.07 +/- 1016.09	1676.62 +/- 971.73	1806.03 +/- 1020.33	1737.22 +/- 1055.42	0.4602	0.5610
ENSG00000164327	RICTOR	mTOR and upstream pathways	575	1609.10 +/- 559.36	1559.79 +/- 607.14	1614.41 +/- 589.17	1652.86 +/- 471.29	0.2626	0.1036
ENSG00000179632	MAF1	mTOR and upstream pathways	575	6439.06 +/- 2022.31	6289.21 +/- 2075.41	6591.46 +/- 2085.01	6435.71 +/- 1900.74	0.3437	0.4801
ENSG00000274070	CASTOR2	mTOR and upstream pathways	575	624.02 +/- 290.57	600.51 +/- 306.78	626.07 +/- 289.40	645.34 +/- 274.48	0.3183	0.1313
ENSG00000196455	PIK3R4	mTOR and upstream pathways	575	520.77 +/- 165.13	519.98 +/- 182.79	520.69 +/- 170.20	521.62 +/- 140.49	0.9953	0.9226
ENSG00000175224	ATG13	mTOR and upstream pathways	575	1008.31 +/- 316.65	993.52 +/- 337.78	1029.98 +/- 339.91	1001.35 +/- 267.51	0.4953	0.8104
ENSG00000159720	ATP6V0D1	mTOR and upstream pathways	575	1823.63 +/- 626.31	1859.93 +/- 668.30	1860.18 +/- 637.24	1750.97 +/- 566.13	0.1438	0.0884
ENSG00000110719	TCIRG1	mTOR and upstream pathways	575	266.60 +/- 125.12	268.14 +/- 146.76	278.04 +/- 128.33	253.63 +/- 94.07	0.1575	0.2556
ENSG00000185344	ATP6V0A2	mTOR and upstream pathways	575	486.66 +/- 151.88	469.06 +/- 163.60	488.73 +/- 155.61	502.09 +/- 133.91	0.1010	0.0332
ENSG00000119655	NPC2	mTOR and upstream pathways	575	490.66 +/- 162.80	491.49 +/- 166.05	497.40 +/- 176.49	483.11 +/- 144.82	0.6894	0.6145
ENSG00000105929	ATP6V0A4	mTOR and upstream pathways	575	0.62 +/- 0.97	0.71 +/- 1.00	0.66 +/- 0.99	0.50 +/- 0.92	0.0864	0.0327
ENSG00000185883	ATP6V0C	mTOR and upstream pathways	575	7.06 +/- 3.77	6.85 +/- 3.81	7.58 +/- 3.75	6.73 +/- 3.72	0.0561	0.7447
ENSG00000141458	NPC1	mTOR and upstream pathways	575	301.72 +/- 104.74	297.40 +/- 111.09	308.24 +/- 111.02	299.51 +/- 91.12	0.5624	0.8458
ENSG00000100554	ATP6V1D	mTOR and upstream pathways	575	1295.00 +/- 367.78	1273.90 +/- 385.56	1305.44 +/- 384.59	1305.56 +/- 331.71	0.6253	0.4004
ENSG00000116039	ATP6V1B1	mTOR and upstream pathways	575	11.60 +/- 8.29	11.16 +/- 7.71	11.81 +/- 8.76	11.83 +/- 8.40	0.6634	0.4257
ENSG00000033627	ATP6V0A1	mTOR and upstream pathways	575	2940.20 +/- 981.92	3001.28 +/- 1096.04	2904.26 +/- 1006.45	2915.38 +/- 827.00	0.5723	0.3929
ENSG00000177058	SLC38A9	mTOR and upstream pathways	575	184.84 +/- 56.18	184.27 +/- 60.26	186.94 +/- 58.93	183.31 +/- 48.93	0.8065	0.8660
ENSG00000171130	ATP6V0E2	mTOR and upstream pathways	575	314.36 +/- 128.72	300.61 +/- 124.15	316.51 +/- 136.23	325.90 +/- 124.79	0.1514	0.0545
ENSG00000149357	LAMTOR1	mTOR and upstream pathways	575	1541.92 +/- 478.29	1541.88 +/- 517.86	1559.23 +/- 469.45	1524.64 +/- 446.91	0.7785	0.7239
ENSG00000128524	ATP6V1F	mTOR and upstream pathways	575	1288.83 +/- 383.59	1284.42 +/- 398.52	1299.66 +/- 399.82	1282.38 +/- 352.40	0.8906	0.9580
ENSG00000117410	ATP6V0B	mTOR and upstream pathways	575	1206.97 +/- 389.85	1196.98 +/- 402.23	1233.59 +/- 401.86	1190.27 +/- 365.01	0.5041	0.8649
ENSG00000113732	ATP6V0E1	mTOR and upstream pathways	575	1240.75 +/- 367.17	1259.15 +/- 390.65	1237.37 +/- 370.35	1225.82 +/- 340.02	0.6667	0.3751
ENSG00000213760	ATP6V1G2	mTOR and upstream pathways	575	22.94 +/- 9.52	23.16 +/- 9.82	23.42 +/- 10.30	22.24 +/- 8.32	0.4487	0.3480
ENSG00000155097	ATP6V1C1	mTOR and upstream pathways	575	759.82 +/- 224.74	751.00 +/- 246.99	763.28 +/- 237.98	765.15 +/- 185.36	0.8000	0.5385
ENSG00000143882	ATP6V1C2	mTOR and upstream pathways	575	15.02 +/- 10.49	16.59 +/- 12.43	14.36 +/- 9.90	14.11 +/- 8.68	0.0394	0.0208
ENSG00000250565	ATP6V1E2	mTOR and upstream pathways	575	164.85 +/- 60.96	162.77 +/- 59.92	166.30 +/- 67.24	165.47 +/- 55.39	0.8398	0.6657
ENSG00000182220	ATP6AP2	mTOR and upstream pathways	575	625.97 +/- 191.58	631.65 +/- 203.72	635.21 +/- 203.82	611.09 +/- 164.73	0.4128	0.2935
ENSG00000114573	ATP6V1A	mTOR and upstream pathways	575	521.56 +/- 164.19	526.45 +/- 181.96	524.54 +/- 174.20	513.73 +/- 132.80	0.7166	0.4489
ENSG00000131100	ATP6V1E1	mTOR and upstream pathways	575	2963.56 +/- 797.38	2952.88 +/- 834.03	2976.09 +/- 819.64	2961.65 +/- 739.42	0.9596	0.9148
ENSG00000047249	ATP6V1H	mTOR and upstream pathways	575	1014.40 +/- 287.45	1028.91 +/- 313.80	1021.89 +/- 296.55	992.47 +/- 248.34	0.4209	0.2149
ENSG00000147416	ATP6V1B2	mTOR and upstream pathways	575	2192.60 +/- 642.26	2168.91 +/- 695.12	2185.83 +/- 664.89	2222.93 +/- 561.60	0.7021	0.4108
ENSG00000136888	ATP6V1G1	mTOR and upstream pathways	575	1971.75 +/- 546.82	1963.83 +/- 601.47	1972.42 +/- 545.13	1978.97 +/- 491.38	0.9639	0.7867

Data shown as n(%), mean +/- SD.

P-values for continuous variables from ANOVA for normally distributed data.

P for linear trend across categories was calculated with linear regression models for those normally distributed variables.

Supplemental Table 3

Gene_symbol	ensembl_gene_id	Base Mean	Log 2-Fold Change	Unadjusted p-value	FDR adjusted p-value	Pathways
AKT1	ENSG00000142208	1679.105975	0.024064654	0.002448793	0.012163011	mTOR and upstream pathways
ATF2	ENSG00000115966	847.7575134	-0.024972539	0.009809844	0.036848228	Autophagy regulators
ATP6V0A1	ENSG00000033627	2693.501901	-0.02782783	0.010184733	0.03788403	mTOR and upstream pathways
ATP6V0A2	ENSG00000185344	451.1931943	-0.033906531	0.000245453	0.001843963	mTOR and upstream pathways
ATP6V1B1	ENSG00000116039	10.82917606	0.122641826	0.003267728	0.014991637	mTOR and upstream pathways
ATP6V1D	ENSG00000100554	1217.944632	0.046659395	5.81E-08	1.40E-06	mTOR and upstream pathways
BMT2	ENSG00000164603	193.2253747	-0.049217188	6.86E-06	9.37E-05	mTOR and upstream pathways
BNIP3L	ENSG00000104765	708.3818075	-0.039051841	0.002029025	0.010696879	Mitophagy
BTRC	ENSG00000166167	950.4500598	0.02247446	0.005245075	0.022043988	mTOR and upstream pathways
CAB39L	ENSG00000102547	322.9893161	-0.147405508	5.54E-08	1.39E-06	mTOR and upstream pathways
CEBPB	ENSG00000172216	4709.343821	-0.050588166	0.00480109	0.020929978	Autophagy regulators
CERS1	ENSG00000223802	86.96219753	-0.077172926	0.000546073	0.003687524	Mitophagy
CRTC2	ENSG00000160741	685.7192548	-0.025302611	0.002771598	0.013220083	Autophagy regulators
DAP	ENSG00000112977	173.5287431	-0.055419422	0.004294704	0.019119386	mTOR and upstream pathways
DDIT3	ENSG00000175197	411.7086255	0.068028418	2.07E-05	0.000235005	Autophagy regulators
DNM1L	ENSG00000087470	2487.381447	0.068482157	1.72E-10	9.41E-09	Mitophagy
E2F1	ENSG00000101412	48.68966971	-0.070083933	5.38E-05	0.000538892	Autophagy regulators
EEF2K	ENSG00000103319	3124.449187	-0.100350519	5.38E-13	4.04E-11	mTOR and upstream pathways
EGR1	ENSG00000120738	431.9868046	-0.235826919	5.60E-05	0.000546961	Autophagy regulators
EPAS1	ENSG00000116016	2961.399273	0.127409519	7.19E-20	1.08E-17	Autophagy regulators
FNIP2	ENSG00000052795	670.6710003	0.047799284	0.008910824	0.034775358	mTOR and upstream pathways
FOS	ENSG00000170345	567.1402362	-0.330110423	1.62E-05	0.000190601	Autophagy regulators
FOXO1	ENSG00000150907	1529.156829	-0.100185619	6.72E-05	0.000621166	Autophagy regulators
FOXO6	ENSG00000204060	57.18608707	-0.088851586	0.002103141	0.010896448	Autophagy regulators
FUNDC1	ENSG00000069509	260.2788752	0.044220109	1.41E-05	0.000170013	Mitophagy
HDAC5	ENSG00000108840	3496.539245	-0.036793441	0.000394094	0.002722422	mTOR and upstream pathways
HRAS	ENSG00000174775	995.7398619	-0.056042286	0.000992019	0.005788384	mTOR and upstream pathways
HSF1	ENSG00000185122	2731.410166	0.036540468	0.000238152	0.001811765	Autophagy regulators
KPTN	ENSG00000118162	181.9723754	0.050572088	0.000704636	0.004428707	mTOR and upstream pathways
LAMTOR5	ENSG00000134248	2238.399804	0.035640192	0.001763771	0.009464519	mTOR and upstream pathways
LMX1A	ENSG00000162761	13.99438643	-0.073580705	0.01090765	0.040217778	Autophagy regulators
LRPPRC	ENSG00000138095	7946.427633	0.050943452	2.72E-06	4.09E-05	Mitophagy
MAPKAP1	ENSG00000119487	2236.386635	0.034935468	9.71E-06	0.000126906	mTOR and upstream pathways
MCL1	ENSG00000143384	7984.830326	-0.042244347	5.64E-05	0.000546961	Mitophagy
MFF	ENSG00000168958	1675.907203	0.032281902	2.44E-07	4.59E-06	Mitophagy
MFN2	ENSG00000116688	13087.42249	0.086099174	4.16E-11	2.78E-09	Mitophagy
MIEF1	ENSG00000100335	620.1736585	0.024492782	0.000250485	0.001858534	Mitophagy
MRAS	ENSG00000158186	1448.304354	-0.0509581	0.003563752	0.016103871	mTOR and upstream pathways
MTOR	ENSG00000198793	1766.21784	0.038653488	0.001301152	0.007377286	mTOR and upstream pathways
MUL1	ENSG00000090432	949.9611892	0.053827359	2.09E-09	8.37E-08	Mitophagy
MYC	ENSG00000136997	171.6448316	-0.165630156	1.88E-08	5.96E-07	Autophagy regulators
NFE2L1	ENSG00000082641	38693.44682	-0.037873368	0.001232563	0.00712279	Autophagy regulators
NFE2L2	ENSG00000116044	1520.824918	-0.03646144	0.000191782	0.001539012	Autophagy regulators
NFKB1	ENSG00000109320	676.5403093	-0.039249614	3.16E-06	4.62E-05	Autophagy regulators
NIPSNAP2	ENSG00000146729	13123.15542	0.04560939	1.33E-06	2.05E-05	Mitophagy
NRAS	ENSG00000213281	492.4770253	-0.035270018	0.003195378	0.014920407	mTOR and upstream pathways
NRF1	ENSG00000106459	664.5912637	-0.030344984	0.003180367	0.014920407	Autophagy regulators
OPA1	ENSG00000198836	3047.355389	0.062361993	3.48E-08	9.97E-07	Mitophagy
PHB1	ENSG00000167085	2918.083448	0.051871427	5.04E-08	1.32E-06	Mitophagy
PHB2	ENSG00000215021	4493.869429	0.025193202	0.002695566	0.012960281	Mitophagy
PIK3R3	ENSG00000117461	265.6193859	0.093087542	5.23E-07	8.98E-06	mTOR and upstream pathways
PIK3R6	ENSG00000276231	15.42139802	0.105292827	0.00222282	0.01132131	mTOR and upstream pathways
PPARA	ENSG00000186951	2774.521182	0.087555659	1.73E-07	3.59E-06	Autophagy regulators
PPARGC1A	ENSG00000109819	1607.242837	0.164007543	1.57E-21	4.72E-19	Autophagy regulators
PRKAA2	ENSG00000162409	8332.71117	-0.0337775	0.009763991	0.036848228	mTOR and upstream pathways
PRKAG1	ENSG00000181929	2564.974064	0.033670511	0.002338892	0.011812386	mTOR and upstream pathways
PRKAG2	ENSG00000106617	249.353462	0.11523078	4.83E-09	1.71E-07	mTOR and upstream pathways
PRKAG3	ENSG00000115592	2042.829824	-0.209413915	1.07E-13	9.15E-12	mTOR and upstream pathways
PRR5	ENSG00000186654	16.13931361	-0.118679503	0.005225588	0.022043988	mTOR and upstream pathways
RARA	ENSG00000131759	355.5974946	-0.05198989	0.000346543	0.002479432	Autophagy regulators
RELA	ENSG00000173039	975.966911	-0.036195055	3.23E-06	4.62E-05	Autophagy regulators
RELB	ENSG00000104856	111.4926588	-0.10838758	2.11E-07	4.23E-06	Autophagy regulators
RPS6KB1	ENSG00000108443	1425.053896	-0.038758672	0.000800166	0.004857573	mTOR and upstream pathways
SEH1L	ENSG00000085415	610.8420205	-0.019615197	0.008720644	0.034480967	mTOR and upstream pathways
SIAH3	ENSG00000215475	2.345228094	0.216268691	0.005962014	0.024883128	Mitophagy
SIRT2	ENSG00000068903	6710.545645	0.045006992	4.02E-05	0.000417404	Autophagy regulators; Mitophagy
SIRT3	ENSG00000142082	922.2225924	0.08643781	1.71E-16	2.06E-14	Autophagy regulators; Mitophagy
SIRT4	ENSG00000089163	135.7449316	0.084284535	2.65E-09	9.96E-08	Autophagy regulators
SIRT5	ENSG00000124523	1375.059189	0.111344175	4.71E-20	9.43E-18	Autophagy regulators; Mitophagy
SP1	ENSG00000185591	1936.847619	-0.02781496	0.011742723	0.043032782	Autophagy regulators
SPATA18	ENSG00000163071	86.42039959	-0.068851609	0.009280737	0.035734387	Mitophagy
STK11	ENSG00000118046	2917.188388	-0.038700892	0.000110099	0.000945281	mTOR and upstream pathways
STRADB	ENSG00000082146	2452.860267	0.047687223	4.37E-05	0.000445638	mTOR and upstream pathways
TBC1D17	ENSG00000104946	2657.598378	-0.033189418	0.002080108	0.010870827	Mitophagy
TFE3	ENSG00000068323	674.836865	-0.0311962	9.43E-05	0.000846183	mTOR and upstream pathways; Autophagy regulators; Mitophagy
TFEB	ENSG00000112561	1774.095501	-0.072554177	5.83E-05	0.000547389	mTOR and upstream pathways; Autophagy regulators; Mitophagy
TGM2	ENSG00000198959	788.3146667	0.09458208	2.20E-07	4.27E-06	Mitophagy
TP53	ENSG00000141510	212.2710692	-0.06458008	5.52E-07	9.22E-06	Autophagy regulators
TSC1	ENSG00000165699	2040.201525	0.026423646	0.009518994	0.036208324	mTOR and upstream pathways
WDR59	ENSG00000103091	1086.039934	-0.018885188	0.003562648	0.016103871	mTOR and upstream pathways
XBP1	ENSG00000100219	749.8328337	-0.0341456	0.001248606	0.007146783	Autophagy regulators
YY1	ENSG00000100811	2075.80181	-0.024755368	1.26E-05	0.000157733	mTOR and upstream pathways; Autophagy regulators

Supplemental Table 4

Gene_symbol	ensembl_gene_id	Base Mean	Log 2-Fold Change	Unadjusted p-value	FDR adjusted p-value	Pathways
AKT1	ENSG00000142208	1726.446311	0.043997502	0.000315953	0.003062702	mTOR and upstream pathways
ATF5	ENSG00000169136	464.2585072	-0.106102341	0.000678744	0.006088439	Autophagy regulators
ATP13A2	ENSG00000159363	408.5317619	-0.056612068	0.000138408	0.001627353	Mitophagy
ATP6V0A1	ENSG00000033627	2758.497524	-0.054826357	0.000901117	0.007289912	mTOR and upstream pathways
ATP6V1B1	ENSG00000116039	11.06782599	0.249300431	0.000128811	0.001548313	mTOR and upstream pathways
ATP6V1D	ENSG00000100554	1234.040439	0.05726331	2.24E-05	0.00040543	mTOR and upstream pathways
BMT2	ENSG00000164603	195.5898311	-0.045034584	0.008022613	0.038572724	mTOR and upstream pathways
CAB39L	ENSG00000102547	321.0011056	-0.165829081	7.16E-05	0.00095686	mTOR and upstream pathways
CERS1	ENSG00000223802	89.53799465	-0.093939717	0.006661925	0.033645522	Mitophagy
CRTC2	ENSG00000160741	704.9112248	-0.039748882	0.002364129	0.014647853	Autophagy regulators
DNM1L	ENSG00000087470	2518.730069	0.081752022	1.01E-06	2.65E-05	Mitophagy
E2F1	ENSG00000101412	50.36217446	-0.080321862	0.002152614	0.014216713	Autophagy regulators
EEF2K	ENSG00000103319	3195.988705	-0.115616812	1.29E-07	4.07E-06	mTOR and upstream pathways
EGR1	ENSG00000120738	454.8098764	-0.4199992	8.41E-07	2.30E-05	Autophagy regulators
EPAS1	ENSG00000116016	3019.731024	0.172820665	2.00E-15	1.20E-12	Autophagy regulators
FOS	ENSG00000170345	584.4036633	-0.639814382	1.78E-09	1.34E-07	Autophagy regulators
FOXO1	ENSG00000150907	1535.767842	-0.112512727	0.003276814	0.018923479	Autophagy regulators
FUNDC1	ENSG00000069509	263.3109115	0.048337729	0.002359455	0.014647853	Mitophagy
GRB10	ENSG00000106070	3323.338908	0.095049604	0.001754615	0.012120961	mTOR and upstream pathways
HDAC5	ENSG00000108840	3612.528038	-0.087568807	1.94E-08	8.95E-07	mTOR and upstream pathways
HRAS	ENSG00000174775	1019.343076	-0.067394298	0.007541615	0.036849681	mTOR and upstream pathways
ITFG2	ENSG00000111203	597.0592098	-0.038195699	0.010481608	0.046641098	mTOR and upstream pathways
JUN	ENSG00000177606	6255.552209	-0.061218507	0.00595469	0.030587765	Autophagy regulators
LAMTOR5	ENSG00000134248	2273.161945	0.055141014	0.001691594	0.011821492	mTOR and upstream pathways
LARP1	ENSG00000155506	3697.06758	-0.038883309	0.012058943	0.049659141	mTOR and upstream pathways
LRPPRC	ENSG00000138095	8073.115769	0.052240176	0.002329379	0.014647853	Mitophagy
LRRK2	ENSG00000188906	694.5137008	-0.103791875	0.00058654	0.005423244	Mitophagy
MAPKAP1	ENSG00000119487	2283.281511	0.056013024	5.20E-06	0.000107765	mTOR and upstream pathways
MCL1	ENSG00000143384	8113.994277	-0.059833943	0.000125321	0.001539684	Mitophagy
MFF	ENSG00000168958	1702.667277	0.032778315	0.000716911	0.006336231	Mitophagy
MFN2	ENSG00000116688	13460.21798	0.079986256	0.000125532	0.001539684	Mitophagy
MIEF1	ENSG00000100335	633.4430415	0.034132707	0.000898313	0.007289912	Mitophagy
MRAS	ENSG00000158186	1455.670137	-0.096640537	0.000176807	0.001864232	mTOR and upstream pathways
MUL1	ENSG00000090432	971.8647563	0.057739602	6.74E-05	0.000942362	Mitophagy
MYC	ENSG00000136997	172.2558822	-0.182146312	2.34E-05	0.00040543	Autophagy regulators
NFE2L1	ENSG00000082641	39807.93633	-0.046257062	0.009671624	0.044126005	Autophagy regulators
NFE2L2	ENSG00000116044	1541.148432	-0.043362531	0.003742665	0.021220204	Autophagy regulators
NFKB1	ENSG00000109320	690.4006344	-0.068261401	1.63E-07	4.90E-06	Autophagy regulators
NIPSNAP2	ENSG00000146729	13332.8865	0.079463428	1.14E-08	5.71E-07	Mitophagy
NPRL2	ENSG00000114388	690.0649477	-0.0373895	0.005186253	0.02734156	mTOR and upstream pathways
NRF1	ENSG00000106459	676.9034718	-0.050178962	0.000875927	0.007289912	Autophagy regulators
OPA1	ENSG00000198836	3092.863017	0.067552891	0.000180513	0.00187049	Mitophagy
PHB1	ENSG00000167085	2983.318471	0.048518822	0.000963679	0.007620668	Mitophagy
PHB2	ENSG00000215021	4595.871591	0.037102938	0.003133947	0.018286427	Mitophagy
PIK3R3	ENSG00000117461	267.8939933	0.093149973	0.00156151	0.011172233	mTOR and upstream pathways
PIK3R6	ENSG00000276231	15.68091159	0.247283793	2.68E-06	5.98E-05	mTOR and upstream pathways
PPARA	ENSG00000186951	2851.661144	0.131801519	4.71E-07	1.35E-05	Autophagy regulators
PPARG	ENSG00000132170	92.23260736	0.151903918	0.000158983	0.001769418	Autophagy regulators
PPARGC1A	ENSG00000109819	1612.988283	0.15563341	4.91E-08	1.84E-06	Autophagy regulators
PRKAB2	ENSG00000131791	6080.036353	0.056892019	0.009529189	0.044126005	mTOR and upstream pathways
PRKAG1	ENSG00000181929	2610.40396	0.067534413	6.16E-05	0.000881417	mTOR and upstream pathways
PRKAG2	ENSG00000106617	251.9214231	0.176715246	6.96E-09	3.80E-07	mTOR and upstream pathways
PRKAG3	ENSG00000115592	2091.25527	-0.247939667	3.89E-08	1.56E-06	mTOR and upstream pathways
RARA	ENSG00000131759	366.0481247	-0.089697491	4.13E-05	0.000636853	Autophagy regulators
RELA	ENSG00000173039	1000.422729	-0.070685078	2.27E-09	1.52E-07	Autophagy regulators
RELB	ENSG00000104856	113.2371704	-0.116024268	0.000171559	0.001841201	Autophagy regulators
SESN3	ENSG00000149212	576.3615899	-0.134511526	0.000232435	0.002328223	mTOR and upstream pathways
SIAH3	ENSG00000215475	2.401262527	0.412425015	0.000909723	0.007289912	Mitophagy
SIRT2	ENSG00000068903	6891.894035	0.05160602	0.002346251	0.014647853	Autophagy regulators; Mitophagy
SIRT3	ENSG00000142082	945.6339346	0.092007399	6.18E-08	2.18E-06	Autophagy regulators; Mitophagy
SIRT5	ENSG00000124523	1400.861271	0.123102205	3.86E-10	3.87E-08	Autophagy regulators; Mitophagy
SPATA18	ENSG00000163071	88.15904353	-0.199477128	1.29E-06	3.10E-05	Mitophagy
STAT3	ENSG00000168610	4605.378316	-0.08388225	6.10E-05	0.000881417	mTOR and upstream pathways; Autophagy regulators
STK11	ENSG00000118046	3010.056578	-0.048912984	0.00143072	0.010359793	mTOR and upstream pathways
STRADB	ENSG00000082146	2487.392756	0.075343072	3.55E-05	0.000587228	mTOR and upstream pathways
TBC1D17	ENSG00000104946	2741.047254	-0.066712245	6.10E-05	0.000881417	Mitophagy
TFE3	ENSG00000068323	688.7803211	-0.038845	0.000863758	0.007289912	mTOR and upstream pathways; Autophagy regulators; Mitophagy
TFEB	ENSG00000112561	1832.366011	-0.071177529	0.009959421	0.045004599	mTOR and upstream pathways; Autophagy regulators; Mitophagy
TGM2	ENSG00000198959	808.164858	0.093650927	0.001330781	0.009753649	Mitophagy
TP53	ENSG00000141510	215.8556264	-0.095212152	1.29E-06	3.10E-05	Autophagy regulators
VCP	ENSG00000165280	8804.948965	-0.040133961	0.003054581	0.017998068	Mitophagy
WDR24	ENSG00000127580	332.9572558	-0.048017096	0.001048662	0.008020652	mTOR and upstream pathways
WDR59	ENSG00000103091	1110.080188	-0.032883722	0.00078392	0.006828057	mTOR and upstream pathways
YY1	ENSG00000100811	2111.344046	-0.023501521	0.007333011	0.036124093	mTOR and upstream pathways; Autophagy regulators

Supplemental Table 5

Gene_symbol	ensembl_gene_id	Base Mean	Log 2-Fold Change	Unadjusted p-value	FDR adjusted p-value	Pathways
ATF5	ENSG00000169136	463.5398625	-0.07797764	0.000235199	0.006145855	Autophagy regulators
ATP6V0A2	ENSG00000185344	455.4089393	0.028912827	0.002790249	0.027948997	mTOR and upstream pathways
BDNF	ENSG00000176697	58.85971971	-0.310173297	4.41E-05	0.002041068	Autophagy regulators
CAB39L	ENSG00000102547	324.0452668	-0.075374439	0.005492021	0.042866292	mTOR and upstream pathways
DNM1L	ENSG00000087470	2499.749531	0.035919593	0.001684173	0.021232321	Mitophagy
EPAS1	ENSG00000116016	2997.467706	0.045101728	0.00343336	0.031412327	Autophagy regulators
GRB10	ENSG00000106070	3292.022346	0.09872924	1.20E-06	0.000144582	mTOR and upstream pathways
IRF1	ENSG00000125347	284.7462519	-0.105604112	0.000200987	0.006011619	Autophagy regulators
ITFG2	ENSG00000111203	595.7175705	-0.033432257	0.000865702	0.014865348	mTOR and upstream pathways
KPTN	ENSG00000118162	185.6730976	-0.048276782	0.001731161	0.021232321	mTOR and upstream pathways
LRPPRC	ENSG00000138095	8028.902751	0.038385128	0.000833887	0.014740177	Mitophagy
MAPKAP1	ENSG00000119487	2266.565587	0.025168982	0.002311777	0.024375057	mTOR and upstream pathways
MFF	ENSG00000168958	1690.064113	0.019962176	0.002417872	0.025054156	Mitophagy
MRAS	ENSG00000158186	1464.330761	-0.087835845	5.09E-07	9.43E-05	mTOR and upstream pathways
MYC	ENSG00000136997	172.9477499	-0.102310521	0.000608134	0.011789959	Autophagy regulators
NFKB1	ENSG00000109320	689.1836844	-0.025770582	0.003210692	0.030150408	Autophagy regulators
NIPSNAP2	ENSG00000146729	13225.79315	0.042715676	1.30E-05	0.000712216	Mitophagy
OPA1	ENSG00000198836	3073.014511	0.04500248	0.000220059	0.006011619	Mitophagy
OPTN	ENSG00000123240	20863.40141	0.047826322	1.24E-05	0.000712216	Mitophagy
PIK3C2B	ENSG00000133056	1319.227198	0.050055458	0.001457815	0.019912424	mTOR and upstream pathways
PIK3CA	ENSG00000121879	1092.769708	0.038635024	0.003748081	0.032179954	mTOR and upstream pathways
PIK3CB	ENSG00000051382	1031.224049	0.042768478	0.005874193	0.043585061	mTOR and upstream pathways
PIK3R5	ENSG00000141506	31.61735265	-0.111242834	0.005862932	0.043585061	mTOR and upstream pathways
PPARA	ENSG00000186951	2821.371413	0.050175876	0.004883876	0.039136128	Autophagy regulators
PRKAB2	ENSG00000131791	6035.551447	0.048563091	0.000740914	0.013915287	mTOR and upstream pathways
PRKAG2	ENSG00000106617	249.6586497	0.065099677	0.00210619	0.023014918	mTOR and upstream pathways
PRKAG3	ENSG00000115592	2092.330888	-0.089022397	0.003037703	0.029485894	mTOR and upstream pathways
RB1CC1	ENSG00000023287	2703.948308	0.046851358	0.000366203	0.007860277	Mitophagy
RELA	ENSG00000173039	994.4375414	-0.022537315	0.006216058	0.045559154	Autophagy regulators
RELB	ENSG00000104856	113.4948543	-0.083841149	6.80E-05	0.002724973	Autophagy regulators
RFX1	ENSG00000132005	260.3736506	0.059117303	0.000288283	0.006663771	Autophagy regulators
SESN3	ENSG00000149212	583.8833234	-0.065816185	0.006634352	0.046790131	mTOR and upstream pathways
STAT1	ENSG00000115415	1817.310298	-0.162629309	6.27E-07	9.43E-05	Autophagy regulators
TFE3	ENSG00000068323	687.7572981	-0.029660584	0.000266616	0.006631057	mTOR and upstream pathways; Autophagy regulators; Mitophagy
TFEC	ENSG00000105967	11.3796088	-0.160299045	0.000605894	0.011789959	Autophagy regulators; Mitophagy
TP53	ENSG00000141510	215.5926339	-0.042804156	0.001548019	0.020674657	Autophagy regulators
TSC1	ENSG00000165699	2075.462962	0.028483508	0.005638702	0.043446919	mTOR and upstream pathways
WDR24	ENSG00000127580	330.8956559	-0.029314632	0.003606407	0.031412327	mTOR and upstream pathways
WDR59	ENSG00000103091	1105.783933	-0.026692786	5.03E-05	0.002159988	mTOR and upstream pathways
ZEB1	ENSG00000148516	4022.010206	0.03746332	0.004345614	0.036273805	Autophagy regulators

Supplemental Table 6

Gene_symbol	ensembl_gene_id	Base Mean	Log 2-Fold Change	Unadjusted p-value	FDR adjusted p-value	Pathways
ATF3	ENSG00000162772	280.0940069	-0.241415565	0.001229716	0.015082838	Autophagy regulators
ATP13A2	ENSG00000159363	405.9265232	-0.039179306	0.001416724	0.016374056	Mitophagy
ATP6V0D1	ENSG00000159720	1713.57596	-0.034429115	0.006160156	0.04304946	mTOR and upstream pathways
CEBPB	ENSG00000172216	4828.741303	-0.088587057	6.01E-05	0.001968742	Autophagy regulators
CIP2A	ENSG00000163507	36.44284318	0.087721004	0.00083251	0.011118631	mTOR and upstream pathways
DNM1L	ENSG00000087470	2498.418901	0.051115699	0.00024913	0.004917931	Mitophagy
EEF2K	ENSG00000103319	3179.83064	-0.066858937	0.000129022	0.003353698	mTOR and upstream pathways
EIF4EBP1	ENSG00000187840	388.5975048	0.079097419	0.002961154	0.027807086	mTOR and upstream pathways
EPAS1	ENSG00000116016	2997.622024	0.056982454	0.002522457	0.02454675	Autophagy regulators
FOS	ENSG00000170345	591.1383536	-0.420140292	1.09E-05	0.000728248	Autophagy regulators
FOXO1	ENSG00000150907	1533.208747	-0.086729754	0.005946638	0.042046225	Autophagy regulators
FOXO6	ENSG00000204060	58.57102047	-0.121720239	0.000644474	0.00923928	Autophagy regulators
GRB10	ENSG00000106070	3294.039301	0.099076008	5.40E-05	0.001968742	mTOR and upstream pathways
HDAC5	ENSG00000108840	3591.261999	-0.055076493	2.42E-05	0.001299395	mTOR and upstream pathways
HDAC6	ENSG00000094631	1463.365386	-0.052353536	6.89E-07	0.000137988	Mitophagy
JUN	ENSG00000177606	6203.510726	-0.075937933	2.59E-05	0.001299395	Autophagy regulators
LPIN1	ENSG00000134324	5240.589615	0.050483737	0.002244351	0.022480918	mTOR and upstream pathways
MAPK14	ENSG00000112062	1739.407363	-0.025590168	0.006471856	0.043703208	Mitophagy
MAPKAP1	ENSG00000119487	2269.535908	0.035977856	0.000338511	0.005782589	mTOR and upstream pathways
MFF	ENSG00000168958	1689.717739	0.022991716	0.003919621	0.03183368	Mitophagy
MRAS	ENSG00000158186	1453.487454	-0.05765647	0.00574703	0.041582921	mTOR and upstream pathways
NFKB1	ENSG00000109320	687.1637407	-0.032056081	0.003103319	0.028693766	Autophagy regulators
NIPSNAP2	ENSG00000146729	13232.55677	0.052470458	9.31E-06	0.000728248	Mitophagy
NPRL2	ENSG00000114388	685.8091721	-0.033541114	0.003303182	0.028771194	mTOR and upstream pathways
NPRL3	ENSG00000103148	785.5220796	-0.045318994	0.003666851	0.031039122	mTOR and upstream pathways
OPA1	ENSG00000198836	3070.2634	0.045611372	0.002532277	0.02454675	Mitophagy
PHB2	ENSG00000215021	4561.298262	0.032552957	0.001844748	0.020158067	Mitophagy
PIK3C2A	ENSG00000011405	616.7243486	0.053367217	0.003263944	0.028771194	mTOR and upstream pathways
PIK3CB	ENSG00000051382	1029.546844	0.056940937	0.002189002	0.022298138	mTOR and upstream pathways
PPARGC1A	ENSG00000109819	1597.662696	0.096079354	6.86E-05	0.001968742	Autophagy regulators
PRKAA1	ENSG00000132356	1510.557298	0.048214232	0.003889118	0.03183368	mTOR and upstream pathways
PRKAG3	ENSG00000115592	2078.55054	-0.101098958	0.003765711	0.031433228	mTOR and upstream pathways
RB1CC1	ENSG00000023287	2703.332925	0.055162481	0.000567632	0.008528678	Mitophagy
RELA	ENSG00000173039	993.4319795	-0.040955931	4.35E-05	0.0018687	Autophagy regulators
RICTOR	ENSG00000164327	1518.563965	0.064349055	0.00025367	0.004917931	mTOR and upstream pathways
SIRT5	ENSG00000124523	1385.420195	0.045904177	0.005386144	0.039476491	Autophagy regulators; Mitophagy
SPATA18	ENSG00000163071	88.08943635	-0.106622428	0.001346398	0.015866375	Mitophagy
SREBF1	ENSG00000072310	1119.721681	-0.093748404	0.003410718	0.029283453	Mitophagy
STAT3	ENSG00000168610	4558.10197	-0.0699767	3.78E-05	0.001747824	mTOR and upstream pathways; Autophagy regulators
STK11	ENSG00000118046	2997.949416	-0.045674836	0.000292475	0.005326596	mTOR and upstream pathways
STRADB	ENSG00000082146	2472.085665	0.053152831	0.000396168	0.006265708	mTOR and upstream pathways
TBC1D17	ENSG00000104946	2723.753215	-0.048938001	0.000346378	0.005782589	Mitophagy
TELO2	ENSG00000100726	651.0881786	-0.047830726	0.000390364	0.006265708	mTOR and upstream pathways
TFE3	ENSG00000068323	686.6862279	-0.052040256	1.23E-07	3.69E-05	mTOR and upstream pathways; Autophagy regulators; Mitophagy
TFEB	ENSG00000112561	1811.58581	-0.06843106	0.001708827	0.019018617	mTOR and upstream pathways; Autophagy regulators; Mitophagy
TP53	ENSG00000141510	214.7273095	-0.050029398	0.002027107	0.021005023	Autophagy regulators
TSC2	ENSG00000103197	2231.824258	-0.049487118	6.13E-05	0.001968742	mTOR and upstream pathways
TTI1	ENSG00000101407	499.8450235	-0.032590072	0.001114391	0.01424998	mTOR and upstream pathways
UBE2L3	ENSG00000185651	1804.134751	-0.026098836	0.001994907	0.021005023	Mitophagy
USP15	ENSG00000135655	3244.941343	0.039322795	0.004816641	0.036643054	Mitophagy
USP35	ENSG00000118369	159.7704789	0.066487553	0.000546378	0.00841983	Mitophagy
USP8	ENSG00000138592	1040.99752	0.028926181	0.004582662	0.035309996	Mitophagy
VCP	ENSG00000165280	8752.810908	-0.039940899	0.000286125	0.005326596	Mitophagy
WDR24	ENSG00000127580	330.8888665	-0.038517001	0.001655697	0.018774983	mTOR and upstream pathways
WDR59	ENSG00000103091	1102.128607	-0.035012517	1.01E-05	0.000728248	mTOR and upstream pathways

Supplemental Table 7

Gene_symbol	ensembl_gene_id	Base Mean	Log 2-FoldvChange	Unadjusted p-value	FDR adjusted p-value	Pathways
ATF3	ENSG00000162772	280.0940069	-0.304742051	8.95E-05	0.002396176	Autophagy regulators
ATP13A2	ENSG00000159363	405.9265232	-0.041124917	0.001556653	0.019490598	Mitophagy
ATP6V0C	ENSG00000185883	6.474300834	0.110662817	0.004498916	0.034734552	mTOR and upstream pathways
BDNF	ENSG00000176697	58.19065228	-0.332470334	0.002321391	0.025226043	Autophagy regulators
CIP2A	ENSG00000163507	36.44284318	0.098243913	0.000465819	0.007367291	mTOR and upstream pathways
E2F1	ENSG00000101412	49.88540517	-0.071753356	0.001636815	0.019874476	Autophagy regulators
EEF2K	ENSG00000103319	3179.83064	-0.054828779	0.003027388	0.029827217	mTOR and upstream pathways
EGR1	ENSG00000120738	451.2638197	-0.280823516	0.000433848	0.007047106	Autophagy regulators
EPAS1	ENSG00000116016	2997.622024	0.074873807	0.000138961	0.0030115	Autophagy regulators
FOS	ENSG00000170345	591.1383536	-0.353699821	0.000231667	0.004491347	Autophagy regulators
FOXO6	ENSG00000204060	58.57102047	-0.154150443	2.90E-05	0.001024425	Autophagy regulators
GRB10	ENSG00000106070	3294.039301	0.129612126	5.04E-07	0.000100915	mTOR and upstream pathways
HDAC5	ENSG00000108840	3591.261999	-0.040800238	0.003349071	0.032376509	mTOR and upstream pathways
HDAC6	ENSG00000094631	1463.365386	-0.049093406	1.28E-05	0.000613581	Mitophagy
JUN	ENSG00000177606	6203.510726	-0.062821787	0.000998916	0.013051055	Autophagy regulators
LPIN1	ENSG00000134324	5240.589615	0.07726166	5.54E-06	0.000555205	mTOR and upstream pathways
MAPKAP1	ENSG00000119487	2269.535908	0.043953859	2.54E-05	0.000953669	mTOR and upstream pathways
MCL1	ENSG00000143384	8063.652316	-0.037549277	0.006109791	0.041727097	Mitophagy
MFF	ENSG00000168958	1689.717739	0.032347043	9.57E-05	0.002396176	Mitophagy
MIEF1	ENSG00000100335	628.9306237	0.027635812	0.001981282	0.022466987	Mitophagy
MYC	ENSG00000136997	171.5258298	-0.148237772	9.14E-05	0.002396176	Autophagy regulators
NFKB1	ENSG00000109320	687.1637407	-0.034755647	0.002421553	0.025446744	Autophagy regulators
NIPSNAP2	ENSG00000146729	13232.55677	0.054738761	7.76E-06	0.000561542	Mitophagy
NPRL2	ENSG00000114388	685.8091721	-0.043449324	0.000277228	0.005206689	mTOR and upstream pathways
NPRL3	ENSG00000103148	785.5220796	-0.048221051	0.003916499	0.034113278	mTOR and upstream pathways
PHB2	ENSG00000215021	4561.298262	0.031876565	0.003617606	0.032450462	Mitophagy
PIK3C2B	ENSG00000133056	1314.182184	0.075745992	0.000106065	0.002549799	mTOR and upstream pathways
PIK3CB	ENSG00000051382	1029.546844	0.067622301	0.000517478	0.007974474	mTOR and upstream pathways
PPP2CA	ENSG00000113575	2626.204079	-0.048671443	0.004845163	0.036122047	mTOR and upstream pathways
PRKAG2	ENSG00000106617	249.8758018	0.07157841	0.007398435	0.048180733	mTOR and upstream pathways
PRKN	ENSG00000185345	1069.759711	0.060182641	0.000217429	0.004355823	Mitophagy
RB1CC1	ENSG00000023287	2703.332925	0.049185255	0.003563935	0.032450462	Mitophagy
RELA	ENSG00000173039	993.4319795	-0.041269985	9.46E-05	0.002396176	Autophagy regulators
SOX2	ENSG00000181449	1.706577973	-0.254874131	0.00434275	0.034734552	Autophagy regulators; Mitophagy
SPATA18	ENSG00000163071	88.08943635	-0.13765801	7.38E-05	0.002217945	Mitophagy
SREBF1	ENSG00000072310	1119.721681	-0.096198456	0.004565773	0.034734552	Mitophagy
STAT1	ENSG00000115415	1801.656599	-0.117752781	0.005143768	0.036369468	Autophagy regulators
STAT3	ENSG00000168610	4558.10197	-0.064358169	0.000355059	0.006257578	mTOR and upstream pathways; Autophagy regulators
STRADB	ENSG00000082146	2472.085665	0.069169967	9.12E-06	0.000561542	mTOR and upstream pathways
TBC1D17	ENSG00000104946	2723.753215	-0.041757399	0.004280385	0.034734552	Mitophagy
TELO2	ENSG00000100726	651.0881786	-0.045921088	0.001385009	0.017710429	mTOR and upstream pathways
TFE3	ENSG00000068323	686.6862279	-0.044734219	1.71E-05	0.000735162	mTOR and upstream pathways; Autophagy regulators; Mitophagy
TSC2	ENSG00000103197	2231.824258	-0.047032619	0.000364418	0.006257578	mTOR and upstream pathways
TTI1	ENSG00000101407	499.8450235	-0.031678424	0.00254044	0.025446744	mTOR and upstream pathways
WDR24	ENSG00000127580	330.8888665	-0.039453389	0.002350513	0.025226043	mTOR and upstream pathways
WDR59	ENSG00000103091	1102.128607	-0.037154565	8.95E-06	0.000561542	mTOR and upstream pathways

Supplemental Table 8

Gene_symbol	ensembl_gene_id	Base Mean	Log 2-Fold Change	Unadjusted p-value	FDR adjusted p-value	Pathways
ATF3	ENSG00000162772	280.6067614	-0.373579205	0.005037579	0.036042676	Autophagy regulators
ATP13A2	ENSG00000159363	406.8873532	-0.060315062	0.007207061	0.043751959	Mitophagy
ATP6V0A1	ENSG00000033627	2740.790157	-0.06560351	0.00830109	0.047970724	mTOR and upstream pathways
ATP6V0E2	ENSG00000171130	294.0610465	0.094165429	0.004164986	0.031289454	mTOR and upstream pathways
ATP6V1D	ENSG00000100554	1227.391004	0.055135416	0.006124324	0.040380398	mTOR and upstream pathways
BTRC	ENSG00000166167	965.1038874	0.048499319	0.008013016	0.047523391	mTOR and upstream pathways
CIP2A	ENSG00000163507	36.60993966	0.166096683	0.000843242	0.00938497	mTOR and upstream pathways
CRTC2	ENSG00000160741	701.2455514	-0.057632891	0.003172356	0.025086661	Autophagy regulators
DNM1L	ENSG00000087470	2500.877005	0.108616584	1.63E-05	0.000376082	Mitophagy
EEF2K	ENSG00000103319	3196.501445	-0.107326642	0.00093713	0.010057418	mTOR and upstream pathways
EGR1	ENSG00000120738	453.9589839	-0.608612355	2.91E-06	0.000125106	Autophagy regulators
EPAS1	ENSG00000116016	3001.617861	0.152283041	6.10E-06	0.000203822	Autophagy regulators
ESR2	ENSG00000140009	21.89570322	0.197278144	0.00145653	0.014118943	Autophagy regulators
FOS	ENSG00000170345	592.2798074	-1.005281248	1.60E-09	4.81E-07	Autophagy regulators
FOXO1	ENSG00000150907	1547.372201	-0.223245413	7.15E-05	0.001301445	Autophagy regulators
HDAC5	ENSG00000108840	3597.947023	-0.1338381	1.42E-08	2.84E-06	mTOR and upstream pathways
HDAC6	ENSG00000094631	1470.184469	-0.08663924	6.76E-06	0.000213693	Mitophagy
HSPA1L	ENSG00000204390	203.7377419	-0.118922822	0.002820062	0.02303565	Mitophagy
ITFG2	ENSG00000111203	595.9601499	-0.084257601	0.000145285	0.002359894	mTOR and upstream pathways
JUN	ENSG00000177606	6231.799497	-0.09376118	0.004634635	0.033968486	Autophagy regulators
JUNB	ENSG00000171223	276.1250466	-0.256524497	0.004756297	0.034440177	Autophagy regulators
LGALS8	ENSG00000116977	407.6079835	0.0739783	0.005616551	0.03879939	mTOR and upstream pathways
MAPKAP1	ENSG00000119487	2265.481652	0.097418352	7.47E-08	9.00E-06	mTOR and upstream pathways
MUL1	ENSG00000090432	965.8098573	0.073809647	0.000641448	0.008031464	Mitophagy
NFE2L2	ENSG00000116044	1538.159603	-0.06374843	0.003673527	0.027946709	Autophagy regulators
NFKB1	ENSG00000109320	690.2515326	-0.07920948	4.29E-05	0.000860059	Autophagy regulators
NIPSNAP2	ENSG00000146729	13231.56623	0.077351803	0.000434908	0.006223322	Mitophagy
NPRL2	ENSG00000114388	687.4540899	-0.071633543	0.000477862	0.006651807	mTOR and upstream pathways
NPRL3	ENSG00000103148	787.7355924	-0.095298154	0.000733965	0.008722676	mTOR and upstream pathways
NRF1	ENSG00000106459	671.2533578	-0.061993335	0.007111793	0.043614162	Autophagy regulators
OPA1	ENSG00000198836	3077.113056	0.104548922	0.000101063	0.001735404	Mitophagy
PPARA	ENSG00000186951	2831.80164	0.115362884	0.003666197	0.027946709	Autophagy regulators
PRKAB2	ENSG00000131791	6051.731084	0.098786619	0.002061462	0.019358412	mTOR and upstream pathways
PRKAG1	ENSG00000181929	2587.226019	0.109409016	1.47E-05	0.000367945	mTOR and upstream pathways
PRKAG2	ENSG00000106617	250.022125	0.157723294	0.000740194	0.008722676	mTOR and upstream pathways
PRKAG3	ENSG00000115592	2091.465185	-0.182998719	0.005983712	0.040262539	mTOR and upstream pathways
RARA	ENSG00000131759	365.0281377	-0.089998056	0.006594429	0.042432908	Autophagy regulators
RELA	ENSG00000173039	996.448474	-0.096277938	7.89E-08	9.00E-06	Autophagy regulators
RHEB	ENSG00000106615	1838.571459	-0.054902852	0.005728171	0.039120801	mTOR and upstream pathways; Mitophagy
SEH1L	ENSG00000085415	619.3555115	-0.045709692	0.006739485	0.042636113	mTOR and upstream pathways
SIAH3	ENSG00000215475	2.361772445	0.683076002	0.000115071	0.001921047	Mitophagy
SIRT3	ENSG00000142082	939.4902716	0.102114653	5.10E-05	0.000988734	Autophagy regulators; Mitophagy
SIRT5	ENSG00000124523	1386.21602	0.158558734	8.98E-08	9.00E-06	Autophagy regulators; Mitophagy
SPATA18	ENSG00000163071	88.95826404	-0.27759803	2.76E-06	0.000125106	Mitophagy
SREBF1	ENSG00000072310	1124.626879	-0.24036899	3.29E-05	0.000707109	Mitophagy
STAT3	ENSG00000168610	4576.340045	-0.149437914	1.27E-06	7.62E-05	mTOR and upstream pathways; Autophagy regulators
STK11	ENSG00000118046	3000.486454	-0.093308744	3.96E-05	0.000820082	mTOR and upstream pathways
STRADB	ENSG00000082146	2465.743138	0.138733279	2.81E-07	2.42E-05	mTOR and upstream pathways
TBC1D17	ENSG00000104946	2727.398879	-0.108919009	1.10E-05	0.000300729	Mitophagy
TELO2	ENSG00000100726	652.8724741	-0.064277685	0.008616858	0.049321254	mTOR and upstream pathways
TFE3	ENSG00000068323	688.3606142	-0.079248118	9.70E-06	0.000277703	mTOR and upstream pathways; Autophagy regulators; Mitophagy
TFEB	ENSG00000112561	1817.248698	-0.106150594	0.008296174	0.047970724	mTOR and upstream pathways; Autophagy regulators; Mitophagy
TGM2	ENSG00000198959	800.6433087	0.117549509	0.006934797	0.043230117	Mitophagy
TP53	ENSG00000141510	216.0548137	-0.103881461	0.000498055	0.006651807	Autophagy regulators
USP35	ENSG00000118369	159.7585101	0.101547322	0.004496364	0.033361907	Mitophagy
VCP	ENSG00000165280	8779.220615	-0.054276238	0.006029332	0.040262539	Mitophagy
WDR24	ENSG00000127580	331.9584871	-0.074154682	0.000862485	0.00942461	mTOR and upstream pathways
WDR59	ENSG00000103091	1106.50123	-0.048688092	0.000989335	0.010251556	mTOR and upstream pathways
YY1	ENSG00000100811	2102.462147	-0.034734387	0.008065534	0.047523391	mTOR and upstream pathways; Autophagy regulators
